# A Comprehensive Critical Assessment of Increased Fruit and Vegetable Intake on Weight Loss in Women

**DOI:** 10.3390/nu12071919

**Published:** 2020-06-29

**Authors:** Mark L. Dreher, Nikki A. Ford

**Affiliations:** 1 Nutrition Science Solutions, LLC, 900 S Rainbow Ranch Rd, Wimberley, TX 78676, USA; nss3@sbcglobal.net; 2 Avocado Nutrition Center, 25212 Marguerite Pkwy Ste. 250, Mission Viejo, CA 92692, USA

**Keywords:** women, fruit, vegetables, legumes, weight, body mass index, adiposity, waist circumference, energy density, glycemic load, prospective cohort studies, randomized controlled trials

## Abstract

No previous reviews or meta-analyses have specifically assessed the effects of increased fruit and vegetable (FV) intake on weight loss with a primary focus on women. Several studies show differences between men and women in how increased FV intake affects their weight loss and maintenance, risk of becoming overweight or obese, and the influence of eating speed and frequency on weight control. This analysis provides a comprehensive and visual assessment of the effects of increasing FV intake and long-term weight change from observational studies and weight loss from randomized controlled trials (RCTs) in women. Consistent evidence from prospective studies and RCTs shows that increased intake of FV is a chief contributor to weight loss in women. This effect is enhanced with concurrent dietary restriction of high energy density (ED) or high-fat foods. Yet, the type of FV differentially impacts weight loss in women. Whole FV intake may influence weight through a variety of mechanisms including a reduction in eating rate, providing a satisfying, very-low to low energy density, low glycemic load or low-fat content. Also, FV are the primary source of dietary fiber, which can provide additional support for weight loss in women when consumed at adequate levels.

## 1. Introduction

**Figure A1:**
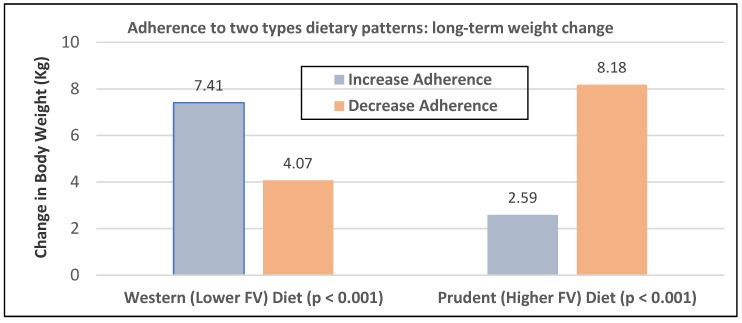
Multivariate adjusted association between dietary pattern adherence and change in body weight from the 51,670 Nurses’ Health Study II women (mean baseline age 26 to 46 years and BMI of 24) over 9-year follow-up [14].

Recent data show that US adults consume inadequate levels of fruits and vegetables (FV). Only 12% of adults meet fruit, and 9% meet vegetable recommendations (e.g., 2 cups of fruits/day and 2.5 cups of vegetables/day based on the 2000-kcal level or ≥5 servings (≥400 g of total FV/day)) with consumption lower among men, young adults, and adults with greater poverty [1,2]. Implementation of the Dietary Guidelines for Americans through the MyPlate approach calls for making FV half of each meal to promote a healthy body weight and protect against chronic diseases [2]. Several studies indicate that there are differences between women and men regarding the impact of increasing FV intake on weight loss and maintenance, or the risk of being overweight or obese [3,4,5]. There also appears to be a sex difference in the influence of eating speed and frequency on weight control. Eight systematic reviews and/or meta-analyses of observational studies and randomized controlled trials (RCTs) are currently published on the effects of increasing FV intake on long-term weight change or weight loss in men, women, and children [6,7,8,9,10,11,12,13]. These reviews and analyses reached a range of conclusions for the effects of increased FV intake on weight loss, including (1) no discernible effects, (2) modest effects, or (3) significant effects. The discrepancies in these conclusions are in part related to the pooling of both men and women, study durations, baseline body mass index (BMI) levels, variations of inclusion and exclusion criteria, differing dietary approaches, anthropometric outcomes measured, eating rates and frequencies, and other factors. None of these publications, specifically or comprehensively examined the effects of increased FV intake on long-term weight change and weight loss focusing on women. One study showed that women who had greater adherence to a Western dietary pattern (low FV) had significantly more weight gain. In contrast, women with increased adherence to a prudent dietary pattern (high FV) had significantly less weight gain (Figure A1) [14]. Therefore, the primary objective of this narrative evaluation is to provide an in-depth assessment of the published prospective cohort studies and RCTs, including the supplemental data, focusing on the unique effects of increased FV intake on body weight in women. Extensive use of figures helps to provide greater clarity and interpretation of the findings. The secondary objectives of this review are to assess small prospective, retrospective, and cross-sectional studies in women to provide additional insights into how FV intake affects weight loss and investigate the various mechanisms involved. The goal of this narrative is to investigate the relationship between FV intake and weight loss but also to provide an extensive and thorough overview of the published literature to inform future research.

## 2. Methods

This comprehensive narrative review included the following search terms: fruit, vegetables, protein foods, legumes, 100% juice, dietary patterns, women, energy density, glycemic load, dietary fiber, weight change, weight gain, weight loss, adiposity, overweight, obesity, abdominal fat, body fat, and waist and hip circumference for searches in PubMed and Google Scholar through 31 May 2020. The term increased FV is equivalent to meeting or exceeding the various dietary guidelines for healthy FV intake or per increased serving. All the papers that met the following criteria were included in this narrative assessment.

Observational studies were subdivided into primary and secondary studies. The primary inclusion criteria included the use of women-only prospective cohort or longitudinal studies or the extraction of data on women only from prospective or longitudinal studies. All primary studies included tables, figures, or supplemental data sets on FV intake and weight loss or other anthropometric changes for analysis. Secondary studies were defined as women-only cross-sectional studies or retrospective studies and one large cross-sectional with 70% women. Secondary studies also included a small prospective study and a large longitudinal study with 63% women. Results are segmented by location from which the research was conducted and by menopause status.

RCTs were subdivided into primary and secondary trials. The primary RCTs only included trials with ≥70% subjects as overweight or obese women randomized to energy-restricted diets (weight loss) or non-energy restricted diets. Only studies with an intervention length of ≥4 weeks were included in the assessment. All primary studies included tables, figures, or supplemental data sets on FV intake and weight loss or other anthropometric changes. Secondary studies were identified as having 60 to 69% women subjects or biological mechanism data sets where no significant interactions between diet type or sex were identified. Dietary approaches and menopause status were used to segment the data.

Secondary findings were explored when discovered in the literature searches described above. Specific mechanisms of action were identified for FV.

## 3. Observational Studies

### 3.1. Primary Studies

Nineteen prospective cohort studies were included that met the primary assessment criteria for the association between increased FV intake and weight change in normal weight and overweight women.

#### 3.1.1. Premenopausal Women

##### Studies from Australia

- Two studies in young Australian women from the Longitudinal Study on Women’s Health (4000 women each; mean baseline age 28 years; BMI 18.5 to <25; and low baseline fruit and vegetable index (FAVI)) showed women in the highest and middle tertile intake of FAVI reduced weight gain by −1.3 to −1.6 kg (95% CI: −0.2 to −2.4 kg) over 6 years (*p* < 0.01 to *p* < 0.05) compared to women in the lowest tertile [15,16]. These studies showed that increased FV intake supported reduced weight gain of 0.12 kg for each 10-point increase in FAVI.

##### Studies from the United States of America

**Figure 1:**
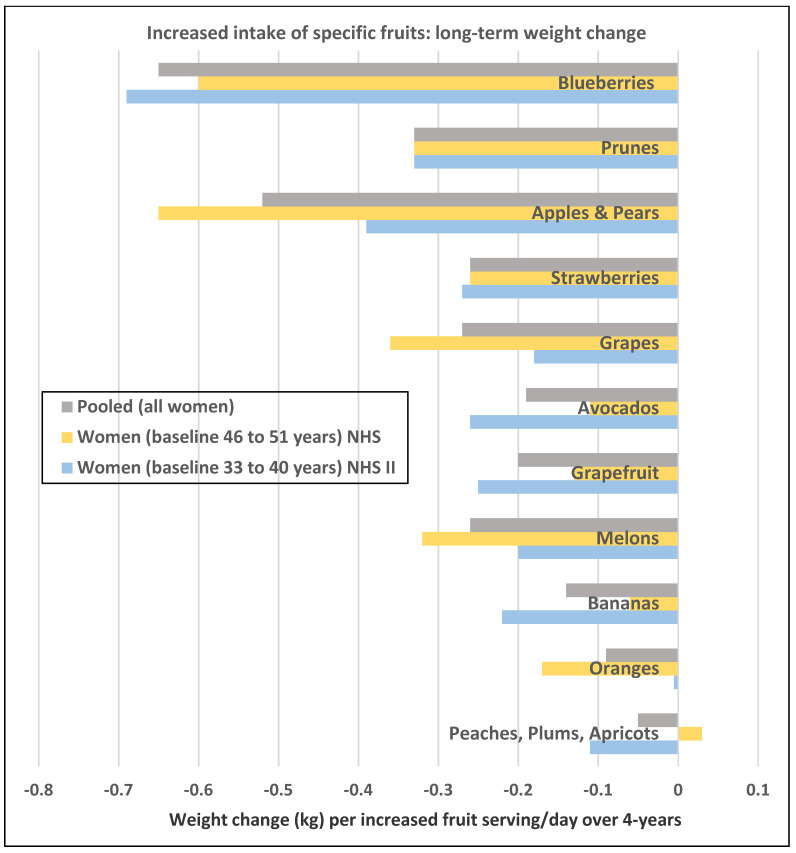
Multivariate adjusted associations from the Nurses’ Health Studies (NHS) per increased serving/day of specific fruits and 4-year weight change (kg) in women of different ages (e.g., premenopausal vs. postmenopausal) [18].

**Figure 2:**
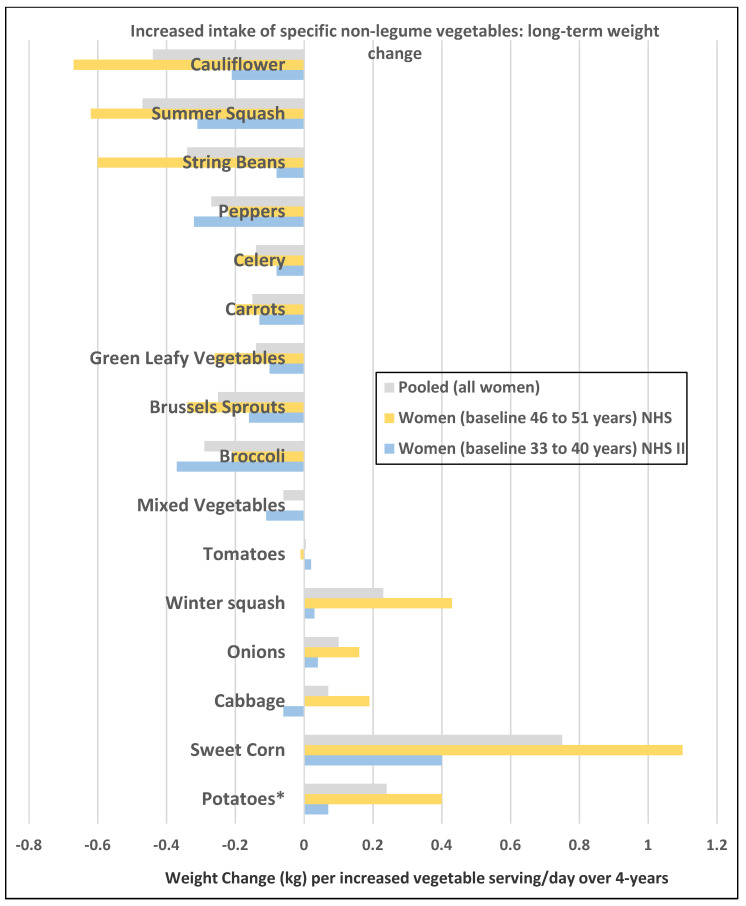
Multivariate adjusted associations from the Nurses’ Health Studies (NHS) per increased serving/day of specific non-legume vegetables and 4-year weight change (kg) in women of different age groups (e.g., premenopausal vs. postmenopausal) [18]. * Includes baked, boiled, and mashed white potatoes, sweet potatoes, and yams but excludes French fries and potato chips.

**Figure 3:**
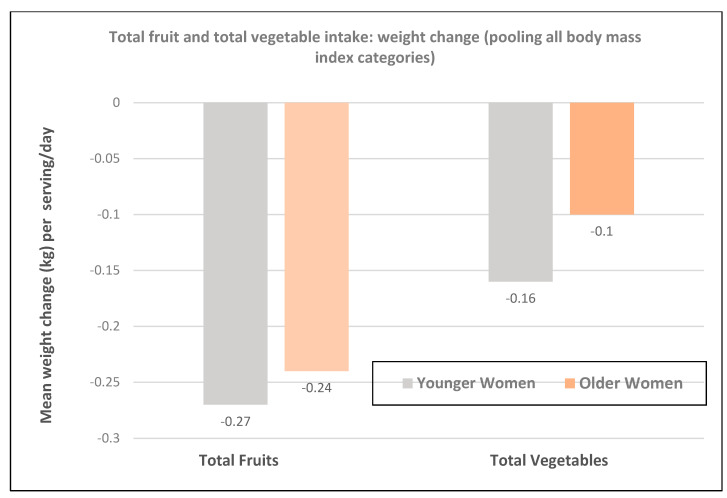
Multivariate association between increased total fruit and total vegetable intake per serving/day and weight change in younger (NHS II; mean baseline age 36-years) and older women (NHS; mean baseline age 49-years) per 4-year period [18].

**Figure 4:**
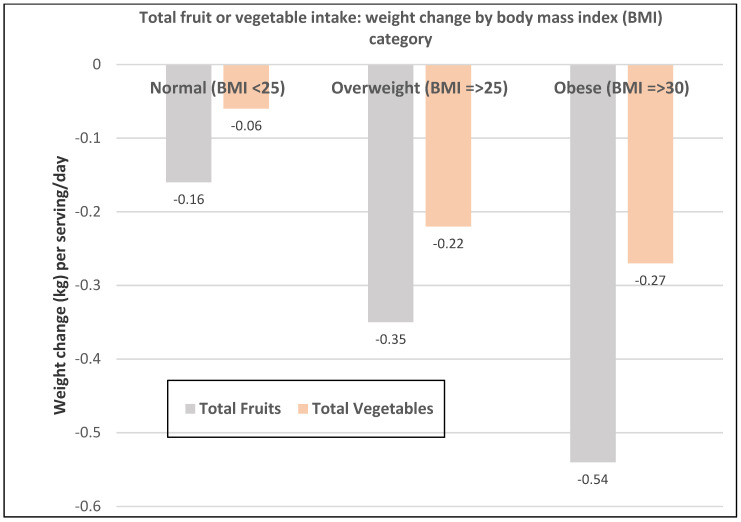
Multivariate adjusted association between total fruit and total vegetable intake per serving/day and pooled mean weight change for all NHS and NHS II women per 4-year period [18].

**Figure 5:**
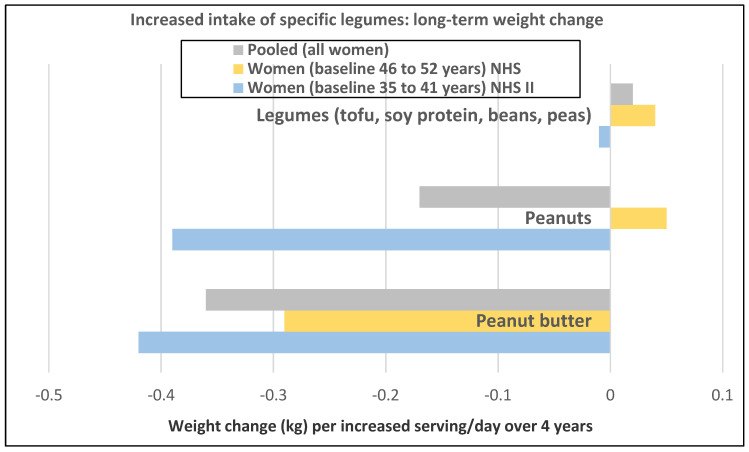
Multivariate adjusted association from 94,922 women in the Nurses’ Health Studies (NHS) per increased serving/day of legumes and 4-year weight change (kg) in women of different age groups [19].

- A prospective study (47,898 NHS II women; mean baseline age of 37.5 and BMI of 23) observed the 4-year weight changes associated with a wide range of foods, including FV and beverages [17]. In premenopausal women mean weight change per daily serving (95% CI; all *p* < 0.001) for total fruit was −0.30 kg (−0.26 to −0.33 kg) and for total vegetables was −0.16 kg (−0.14 to −0.17 kg). French fries increased body weight by +1.65 kg (1.37 to 1.93 kg) and boiled, baked, or mashed potatoes +0.35 kg (0.22 to 0.48 kg). Fruit juice (100%) increased weight by +0.22 kg (0.19 to 0.26 kg).
- A prospective study (73,737 NHS II women; mean baseline age of 36 and BMI of 24.2) observed the mean (95% CI) 4-year weight change per serving/day increase in specific fruits or vegetables summarized in Figure 1 and Figure 2 [18]. In premenopausal women, the mean change in body weight change was −0.27 kg (−0.24 to −0.30 kg) for each total fruit serving/day (95% CI; all *p* < 0.001) and −0.16 kg (−0.14 to −0.17 kg) for each total vegetable serving/day. The top 5 fruits for weight loss were blueberries −0.70 kg (−0.29 to −0.98 kg), apples and pears −0.39 kg (−0.48 to −0.64 kg), prunes −0.33 kg (−0.66 to −0.01 kg), strawberries −0.27 kg (−0.55 to 0.01 kg), and avocados −0.26 kg (−0.79 to 0.28 kg). Whole fruits (excluding fruit juice) were associated with weight loss independent of fiber and GL levels in part because total fruit contains few starchy or high GL varieties. The top 5 non-legume vegetables for weight loss were broccoli −0.37 kg (−0.56 to −0.17), peppers −0.32 kg (−0.57 to −0.06 kg), summer squash −0.31 kg (−0.64 to 0.01 kg), cauliflower −0.21 kg (−0.49 to 0.06 kg), and Brussels sprouts −0.16 kg (−0.61 to 0.28 kg). Total vegetable intake per serving was associated with about half the long-term weight loss compared to total fruit intake. Starchy, lower fiber, and higher GL vegetables were associated with weight gain, and non-starchy vegetables with higher fiber and lower GL were associated with weight loss (*p* < 0.0001). Younger women over four years tended to have greater weight loss than older women per serving of total vegetables (Figure 3) [18], which may be associated with the higher 4-year weight gain observed in postmenopausal women consuming sweet corn, potatoes, cabbage, onions, or winter squash compared to premenopausal women as shown in Figure 2 [18]. Women who were overweight or obese lost more weight than normal-weight women, and increased fruit intake was more strongly associated with 4-year weight loss than vegetable intake (Figure 4) [18].

- A prospective study (47,928 NHS II women; mean baseline age of 38 years and BMI of 23.0) observed the mean (95% CI) 4-year weight change per serving/day increase in legumes (Figure 5) [19]. In NHS II women, legumes (tofu, soy protein, peas, beans, lentils, and lima beans) were associated with no weight change and weight loss was associated with intake of peanuts peanut butter. In another study of premenopausal women, an increase in one tofu and soy serving/day was associated with weight loss of −0.8 kg (−0.5 to −1.2 kg) over 4-years [18]. However, the type of dietary carbohydrates consumed with legumes can influence the trajectory of weight change with higher GL diets reducing the odds of weight loss and lower GL diets increasing the odds of weight loss.

- A longitudinal study of 186 women (mean baseline age of 36 years and BMI of 27) observed that women with higher dietary ED (>1.85 kcal/g) who consumed 2.7 FV servings/day gained an average of about 4 kg more weight (*p* < 0.01) compared to women consuming the lowest dietary ED (<1.5 kcal/g) who consumed 5 FV servings/day over six years [20]. Women consuming the higher FV diet had their mean total energy intake reduced by −225 kcal/day compared to the lower FV diet.

##### Studies from Europe

- A prospective Spanish study of 6613 women (mean baseline age of 43 years and 22 BMI) consuming a Mediterranean diet in the Seguimiento Universidad de Navarra (SUN) Project observed that multivariate-adjusted FV intake was inversely associated with weight gain over five years and higher dietary fiber was directly associated with consumption of FV [21]. Also, in this study, energy-adjusted total dietary fat (olive oil primarily) intake did not support an independent effect of fat on weight gain.

##### Studies from Iran

- In a 3-year longitudinal study, approximately 700 Iranian women (mean baseline age of 40 years) consuming healthy low ED, high fiber, and about 2000 kcal/day diets saw more weight loss with a higher intake of total FV [22]. Red and purple FV were associated with a smaller WC.

#### 3.1.2. Postmenopausal Women

##### Study from New Zealand

- A prospective analysis of the New Zealand Weight-To-Be trial (1293 women; baseline mean age 49.5 years and BMI 34.4) over 24 months observed that hamburgers, fried chicken, hot dogs, bacon or sausage, French fries, and high fat consumption were associated with higher BMI, while eating a diet high in FV, such as green salads, and higher fiber intake were associated with lower BMI [23]. Overall, increasing fruit, vegetables and fiber, and decreasing dietary fat throughout the study were associated with reductions in BMI.

##### Studies from the United States of America

**Figure 6:**
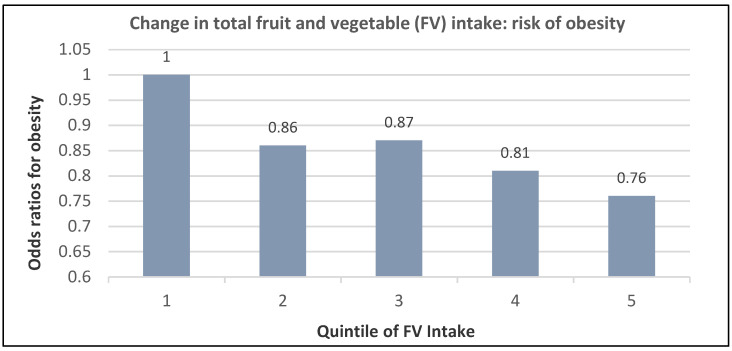
Multivariate adjusted evaluation of total fruit and vegetable intake and risk for obesity over 12 years in women from the Nurses’ Health Study (mean baseline age 50 years and BMI 24.9 (*p*-trend < 0.0001; median change in quintile fruit and vegetable servings from baseline −2.36, −0.49, 0.64, 1.83, and 3.99) [24].

**Figure 7:**
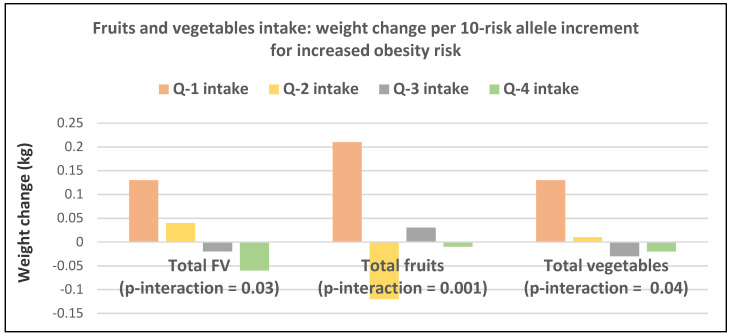
Multivariate adjusted weight change over 4-years per 10 allele increment of genetic susceptibility to obesity for each quintile (Q) of total fruit and vegetable (FV) intake by 8943 middle-aged, overweight women from the Nurses’ Health Study [25].

- A prospective study (74,063 NHS women; mean baseline age 51 years and BMI of 24.9) observed that those in the highest quintile of total fruit intake had a 24% lower risk of becoming obese and a 16% lower risk for those in the highest vegetable intake group compared to women with the lowest intake (*p*-trend < 0.0001) over 12 years of follow-up [24]. The association between increasing quintiles of total FV intake and obesity risk is summarized in Figure 6.

- A prospective study (50,422 NHS women; mean baseline age 52 years and BMI 23.7) observed the 4-year weight changes associated with a wide range of foods, including FV and beverages [17]. In postmenopausal women weight change per daily serving determined at 4-year periods (95% CI); *p* < 0.001 for total fruit was −0.20 kg (−0.17 to −0.22 kg) and for total vegetables was −0.07 kg (−0.06 to −0.09 kg). French fries increased weight by +1.9 kg (1.57 to 2.16 kg) and boiled, baked, or mashed potatoes increased weight by +0.32 kg (0.24 to 0.40 kg). Fruit juice (100%) increased weight by +0.12 kg (0.09 to 0.15 kg).
- A prospective study (40,415 NHS women; mean baseline age 49 years and BMI 24.7) observed the mean (95% CI) 4-year weight change per serving/day increase from specific fruits or vegetables summarized in Figure 1 and Figure 2 [18]. In postmenopausal women, the mean weight change per increased serving/day for total fruit was −0.24 kg (−0.18 to −0.27 kg) and −0.10 kg (−0.08 to −0.12 kg) for total vegetables. The top 5 fruits for weight loss were apples and pears −0.65 kg (−0.56 to −0.74 kg), blueberries −0.60 kg (−0.37 to −0.84 kg), grapes −0.36 kg (−0.24 to −0.47 kg), prunes −0.27 kg (−0.16 to −0.50 kg), and watermelon −0.32 kg (−0.16 to −0.48 kg). Whole fruits (excluding fruit juice) were associated with weight loss independent of fiber and GL levels in part because total fruit contains fewer starchy or high GL varieties. The top 5 non-legume vegetables were cauliflower −0.67 kg (−0.44 to −0.89 kg), summer squash −0.62 kg (−0.37 to −0.85 kg), string beans −0.60 kg (−0.41 to −0.78 kg), Brussels sprouts −0.34 kg (−0.77 to 0.09 kg), and green leafy vegetables −0.26 kg (−0.20 to −0.31 kg). Total vegetable intake per serving was associated with about half the long-term weight loss compared to total fruit [17,18]. Starchy vegetables such as potatoes and sweet corn were associated with weight gain, whereas higher fiber and lower GL vegetables were associated with weight loss (*p* < 0.0001). Older women over four years tended to have less weight loss than younger women per serving of total vegetable intake (Figure 3) [18], which may be associated with the higher 4-year weight gain observed in postmenopausal women consuming sweet corn, potatoes, cabbage, onions or winter squash compared to premenopausal women as shown in Figure 2 [18]. Women who were overweight or obese lost more weight than normal-weight women, and increased fruit intake was more strongly associated with 4-year weight loss than vegetable intake (Figure 4) [18].
- A prospective study (46,994 NHS women; mean baseline age 49 years and BMI 23.7) observed the mean (95% CI) 4-year weight change per serving/day increase in protein foods (Figure 5) [19]. In NHS women, no weight change was associated with legumes (tofu, soy protein, peas, beans, lentils, and lima beans), but weight loss was associated with peanut butter. In another study of postmenopausal women, an increase in a serving of tofu and soy was associated with a loss of −1.3 kg (0.5 to 1.7 kg) over 4-years [18]. However, the type of dietary carbohydrates consumed with legumes can influence the trajectory of weight change with higher GL diets reducing the odds of weight loss and lower GL diets increasing the odds of weight loss [19].
- A prospective study (8943 NHS women; mean baseline age 54 years and BMI of 25.5) examined the effects of total and specific FV intake on genetic susceptibility for increased risk of obesity over repeated 4-year time frames for measuring BMI and body weight changes [25]. BMI change per 10-risk allele increment for susceptibility to obesity was 0.07 + 0.04 kg/m^2^ among women with the highest decrease in total FV intake and −0.02 + 0.03 kg/m^2^ among those with the most substantial increase in FV intake (*p*-interaction < 0.009). Weight change in kg per quartile of FV intake is shown in Figure 7. Berries, citrus fruits, and green leafy vegetables were the most effective in lowering BMI. Women with higher genetic risk for obesity had greater decreased weight with increasing FV intake than those with lower risk.

- The US Women’s Health Initiative (48,106 women; mean baseline age 58 years and BMI of 26.2) observed a loss of −0.43 kg (95% CI: −0.32 to −0.52 kg) for each serving of whole fruit and a weight gain of 0.18 kg (95% CI: 0.05 to 0.31 kg) for each 6 oz serving of 100% fruit juice over three years [26].
- The US Women’s Health Study (18,146 women; mean baseline age 54 years and BMI of 22.4) observed that the consumption of >3.1 fruit servings/day significantly reduced the odds of being overweight or obese by −13% compared to basically no weight change with the consumption of <3 vegetable servings/day (*p*-trend = 0.22) over 15.9 years [27].
- The Stockholm Public Health Cohort (12,860 women; baseline age 48 years and BMI of 24.9) observed that middle-aged women reduced BMI by −0.06, overweight risk by 6% and risk of obesity by 12% with the consumption of at least a daily serving of fruit compared to less than a daily serving over eight years [28].
- The European Prospective into Cancer (EPIC) and Nutrition-Physical Activity, Nutrition, Alcohol, Cessation of Smoking, Eating Out of Home, and Obesity study in a sub-analysis of women (approx.100,000 women; baseline over 50 years and BMI of 25) observed that each 100-g/day of total vegetable intake was associated with an annual weight increase of 6 g/year (*p* = 0.03) and total fruit intake per 100-g/day was associated with an annual weight decrease of −10 g/year (*p* < 0.001) [29].
- EPIC Diet, Obesity and Genes studies (BiOGenes; 28,937 women; baseline age 49 years and BMI of 24.9) over 5.5 years observed in middle-aged normal-weight women that every 100 kcals/day increase in total fruit intake was significantly associated with reduced WC by −11 cm/year compared to −0.05 cm/year for total vegetables whereas every 100 kcals/day of potatoes significantly increased WC by 0.05 cm/year [30,31].
- EPIC BiOGenes study (52,307 women; mean baseline age 53 years and BMI of 25.2) observed in middle-aged slightly overweight women that each 100 g/day increase in FV intake reduced weight gain by −12 g/year (*p* = 0.02) over 6.5 years [32].

##### Studies from China

- The Chinese Health and Nutrition study (2387 women; mean baseline age 46 years and BMI of 23.3) observed in middle-aged normal-weight women that each 100 g/day increase in total FV reduced weight by −0.14 kg and BMI by −0.29 over six years. Still, these reductions did not achieve statistical significance [33]. Despite the study’s finding of an insignificant association with weight loss, the Chinese still recommended an increased FV intake to their population to help protect against long-term risk of weight gain.

### 3.2. Secondary Studies

Seven secondary studies provide additional insights regarding increased FV intake and weight loss or reduced weight gain over time.

#### 3.2.1. Premenopausal Women

- A cross-sectional study of 279 low-income overweight or obese African-American women in Pittsburg, Pennsylvania (mean age of 36 + 9.3 years) found that those consuming >5 FV servings/day were 94% more likely to be weight resilient (maintaining weight stability in an obesity risky environment) compared to those eating <5 servings/day [34].
- A Swedish cross-sectional study of 577 women (mean baseline age of 22 years and BMI of 23) observed that young women consuming the recommended amount of FV (500 g/day) had significantly less % body fat compared to those not consuming the recommended levels of FV (26.5% vs. 28.4% *p* < 0.05), despite no observable differences in BMI or WC between the two groups [35]. This lower % body fat was associated with a significantly lower HOMA-IR (1.48 vs. 1.81; *p* = 0.001), which suggests the importance of increasing FV intake in young women to reduce the potential long-term risk of pre-diabetes or diabetes.

#### 3.2.2. Post-Menopausal Women

**Figure 8:**
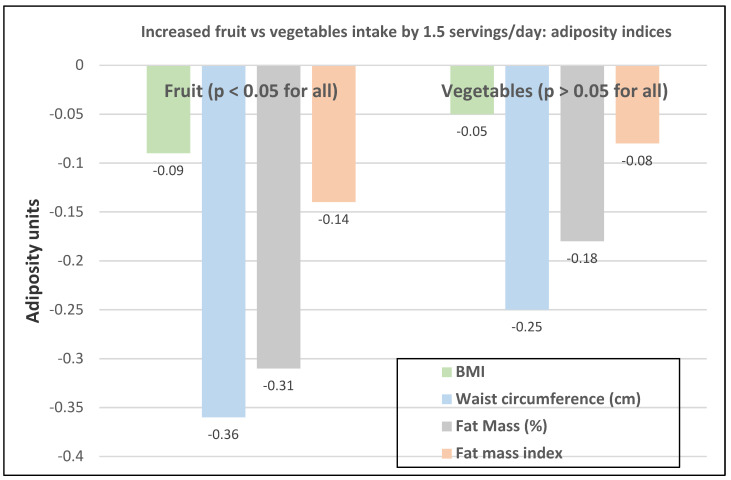
Multivariate adjusted associations between increased intake of fruit and vegetables by 1.5 servings/day and adiposity indices (26,340 participants, 70% women, mean age 53 years, and BMI 28.5) [36].

- The cross-sectional Canadian Atlantic Partnership for Tomorrow’s Health Study (26,340 participants, 70% women, mean baseline age 53) observed that an increase of 2.6 servings of total FV/day was inversely associated with BMI (−0.12 kg/m^2^), WC (−0.4 cm), fat mass (−0.14%), and fat mass index (−0.14 kg/m^2^) [36]. Fruit (1.5 servings) was consistently inversely associated with body adiposity compared with vegetables (1.5 servings) (Figure 8). Fruit (>2 vs. <1 servings/day) reduced risk of abdominal obesity by 12% (*p* < 0.001) and vegetables (>3 vs. <2 servings/day) reduced abdominal obesity risk by 6% (*p* = 0.066).

- In a 6-month retrospective study, 66 obese Brazilian women given guidance to reduce energy intake from ultra-processed foods and increase healthy fiber-rich foods found that women who lost weight increased their consumption of leafy vegetables (*p* = 0.013) and fruit (*p* = 0.004), and reduced sweets (*p* = 0.0008) and fried foods (*p* = 0.01), thus reducing mean body weight by 3.2%, BMI by 2.5 kg/m^2^, body fat mass by 1.7% and WC by 4.8 cm compared to baseline levels [37].
- The Netherlands Epidemiology of Obesity cross-sectional study, including 3576 women (mean baseline age 55 years), observed that visceral fat was reduced by 1.3 cm^2^ for each 100 g increase in fruits or vegetables [38].
- The US and Canadian Adventist Health Study 2 (55,407 normal-weight adults; 63% women; mean baseline age 56-years) observed that the odds of becoming overweight or obese were reduced by 15% in high avocado consumers and 7% in low avocado consumers compared to non-consumers of avocados over an 11-year follow-up [39].
- An Australian cross-sectional study (130,966 women; mean age 62 years) observed that compared to the reference normal-weight women, obese women were 25% and overweight women were 15% less likely to be in the highest fruit intake quartile. In contrast, obese women were 18% and overweight women, 9% more likely to be in the highest vegetable consumption quartile (*p* < 0.01) [3].

### 3.3. Randomized Controlled Trials

#### 3.3.1. Primary Studies

Twelve RCTs met the primary assessment criteria for the impact of FV intake on body weight and other anthropometric measurements in premenopausal women. Another eight RCTs met the primary inclusion criteria for the effects of FV intake on body weight and other anthropometric measurements in postmenopausal women. Results are segmented by different dietary approaches.

**Figure 9:**
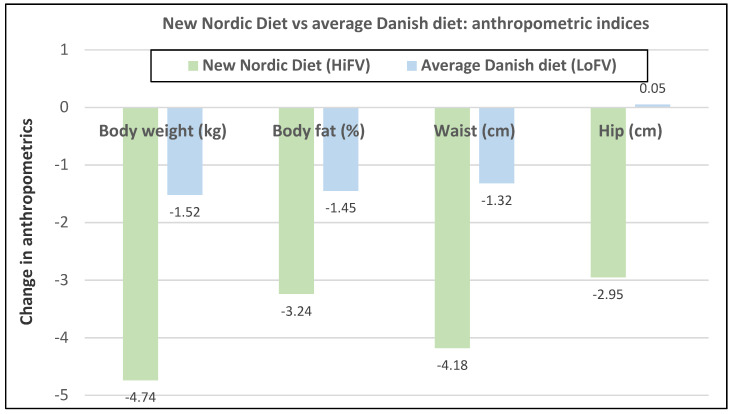
Ad libitum New Nordic Diet (high in healthy fruits and vegetables) vs. average Danish diet (low in fruits and vegetables) in 147 centrally obese adults (71% women; mean age 42 years) who completed the 26-week trial (*p* < 0.001 for all) [40].

**Figure 10:**
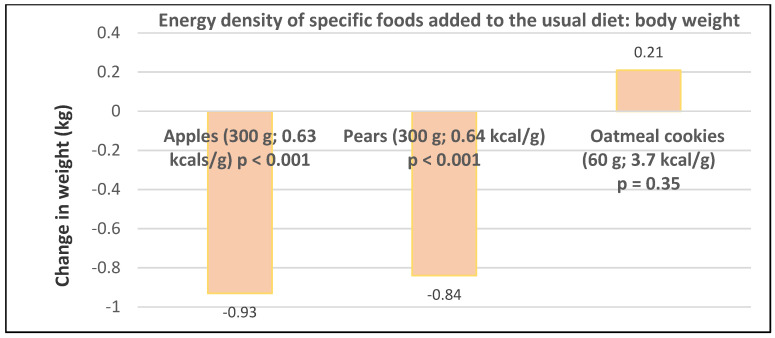
Apples or pears vs. oatmeal cookies added to the usual diet and weight change in 49 overweight and obese women (mean age 44 years) for ten weeks [41].

**Figure 11:**
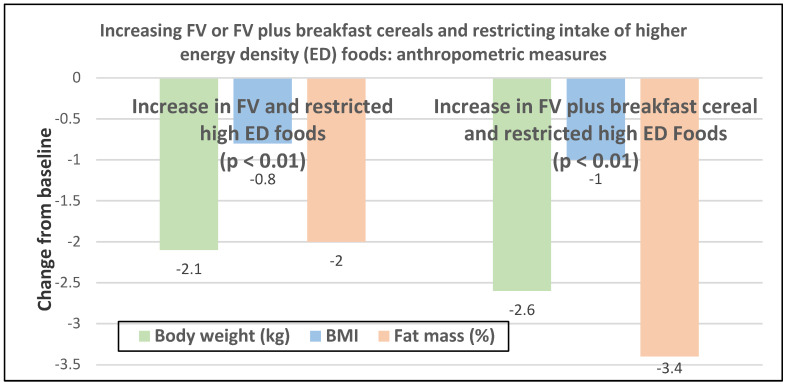
Anthropometric changes with increased intake of fruit and vegetable (FV) or FV plus breakfast cereals, and restricting intake of high energy density foods after six weeks in women (mean baseline age 20–35 years and BMI 28.9) [42].

**Figure 12:**
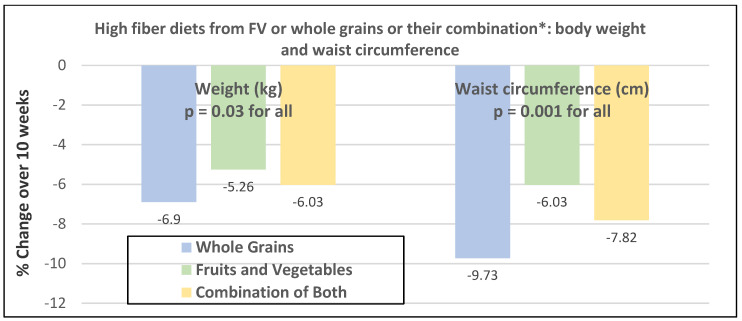
Change in weight and waist circumference in 75 obese women (mean age 37 years) consuming diets rich in fruit and vegetable (FV), whole grains, or a combination of both [45]. * Diets were a mean of 2100 kcals, and all diets contained ≥35 g fiber: 70% fiber from FV or cereals and 50/50% from each in the combination diet.

**Figure 13:**
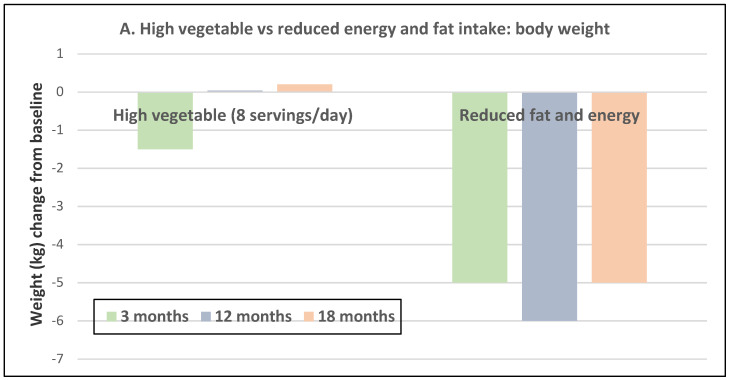

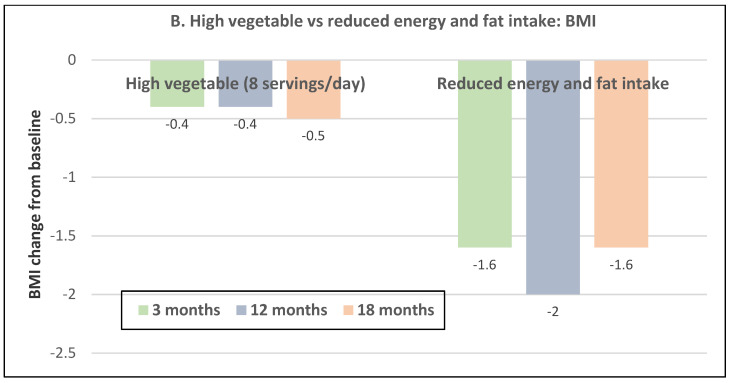
Change in weight and BMI vs. baseline in obese adults (73% obese women; mean age 34 years) for high vegetable (8 servings/day) intake or weight loss diet (500 kcal reduced energy intake) over a three-month intervention and up to 18 months follow-up: (**A**) high vegetable diet reduced weight after three months (*p* = 0.0087) with an increase back to baseline at 12 and 18 months, and reduced energy and fat intake reduced weight at three months (<0.0001), 12 months (*p* = 0.0006), and 18 months (*p* = 0.019), and (**B**) high vegetable diet reduced BMI at three months (*p* = 0.014) which remained reduced for the 15 months of follow-up, and the reduced energy and fat diet reduced BMI which remained reduced for all follow-up periods (*p* ≤ 0.045) [46]. High vegetable group was requested to avoid potato chips, fried vegetables, or 100% fruit or vegetable juices, but post hoc dietary analysis showed that potato chips, French-fried potatoes, 100% vegetable juices were counted as part of the goals and 2–3 servings of fruit including fruit juice.

**Figure 14:**
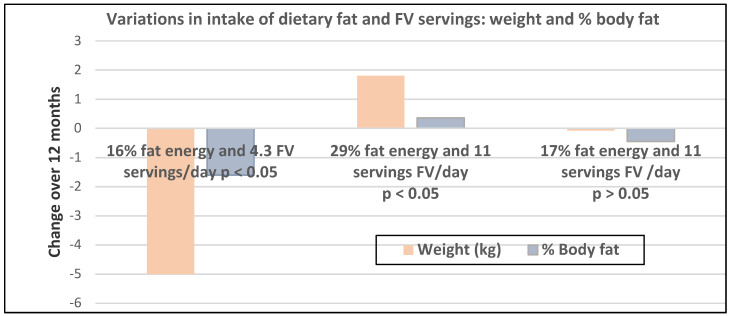
Weight-related changes from baseline with variation in % energy from fat and level of fruit and vegetable (FV) intake, including 100% juice, dried fruit, and starchy vegetables in 122 overweight women (mean age 38 years) over 12 months [47].

**Figure 15:**
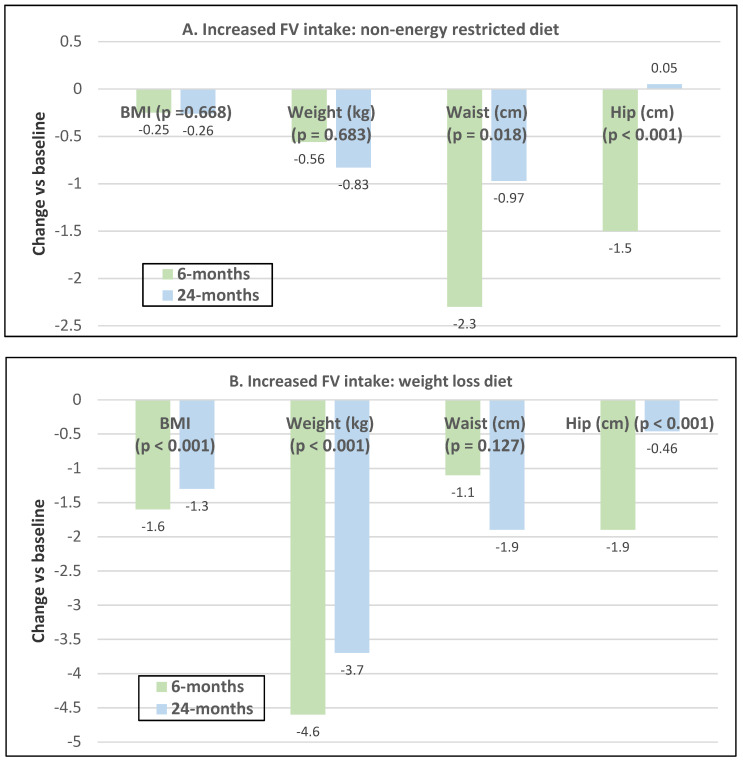
Mean change in anthropometric measures in obese women age 30–45 years: (**A**) increasing fruit and vegetable (FV) intake by 1.3 to 2.2 servings/day with a non-energy restricted diet vs. (**B**) increasing FV intake by 1.3 to 2.2 servings/day with a weight loss diet [48].

**Figure 16:**
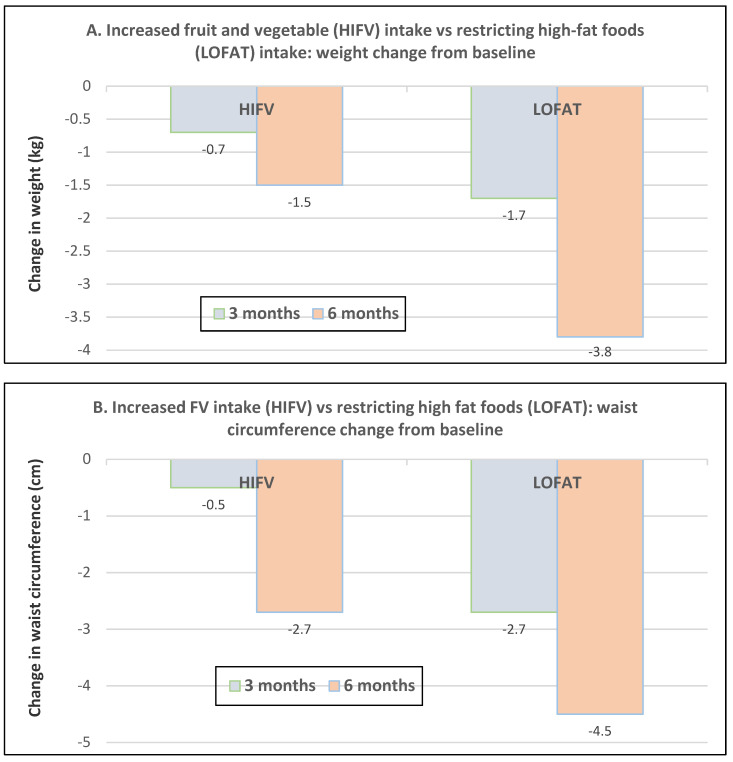
Messaging to encourage increased fruit and vegetable (HIFV) intake vs. restricting intake of high-fat foods (LOFAT) in overweight or obese postmenopausal women: (**A**) weight change (LOFAT group *p* < 0.001 at both 3 and 6 months vs. baseline; HIFV *p* = 0.1829 at three months and *p* = 0.0004 at six months vs. baseline) and (**B**) waist circumference change (both diets *p* < 0.05 at six months vs. baseline) [52].

- Premenopausal women—dietary approach #1—ad libitum diets high in healthy FV and restricting higher ED foods vs. a Western-type diet. Centrally obese adults (71% women; mean baseline age 42 years, BMI of 30, and WC of 100 cm) consuming the New Nordic Diet (high in FV) significantly lowered body weight and fat, and waist and hip circumferences compared to the average Danish diet (low in FV) over 26 weeks (Figure 9) [40]. The New Nordic diet reduced total energy intake by −420 kcals, ED by −0.21 kcal/g, and increased fiber intake by 19 g/day versus the average Danish control (Western) diet. The New Nordic diet significantly reduced both systolic and diastolic blood pressure by −5.1 and −3.2 mmHg, respectively.
- Premenopausal women—dietary approach # 2—adding low ED fruit vs. high ED cookies to the usual diet. Women consuming apples or pears had significant weight loss compared to the modest weight gain of women consuming oatmeal cookies over ten weeks (Figure 10) [41].
- Premenopausal women—dietary approach #3—guidance to increase FV and cereals and restricting the intake of ED rich foods. Overweight women significantly reduced weight, BMI, and/or % body fat from baseline over six weeks when they increased their FV intake by 4.5 servings/day or FV intake by 2.5 servings/day plus one serving of cereal along with concurrently restricting high ED foods (Figure 11) [42]. Similar findings were also reported in two other papers [43,44].
- Premenopausal women—dietary approach #4—increasing high fiber diets from FV and/or cereals with supervision and no energy restrictions. Obese women consuming >35 g/day fiber from FV, whole grain cereals, or a combination of both at high levels with a 2100-kcal diet under supervision, significantly reduced weight and WC after ten weeks (Figure 12) [45].
- Premenopausal women—dietary approach #5—high vegetable diet including fried potatoes, 100% fruit, and vegetable juice vs. diets with 500 kcal reduced energy. Overweight and obese women consuming eight vegetable servings/day significantly reduced weight and BMI over three months with individual consultations and prevented weight regain for 18 months of follow-up, but the 500 kcal restricted diet resulted in additional significant weight loss that was maintained over the 18 months of follow-up (Figure 13A,B) [46].
- Premenopausal women—dietary approach #6—varying percent energy from fat and servings of FV. High FV intake, including 100% juice, dried fruit, and starchy vegetables, reduced weight, and % body fat with 16–17% energy from fat compared to an increase in weight and % body fat with 29% energy from fat (Figure 14) [47].
- Premenopausal women—dietary approach #7—moderate increase in FV intake with weight neutral and weight loss diets. Obese women increasing FV intake by 1.3 to 2.2 servings/day in a weight neutral diet over a 6-month intervention had insignificant BMI and weight reduction and significant reductions in waist and hip measures, which were attenuated toward baseline levels after 18-months of follow-up (24-month time point) whereas those increasing FV in a weight loss diet had significant reductions of BMI, weight, waist and hip circumference over 6-months with little attenuation over the 18-month follow-up (Figure 15A,B) [48].
- Premenopausal women—dietary approach #8—reduced energy weight-loss diets with whole fruit or fruit juice. Obese women (mean age 32 years) who consumed hypocaloric diets had the same weight and body fat % reductions with high or low fruit content over eight weeks [49]. In obese Brazilian adults (approx. 70% women; mean age 36 years and baseline BMI of 33.5), the consumption of orange juice (500 mL/day) did not interfere with the effectiveness of weight-loss diets over 12 weeks [50]. In 51 overweight and obese adults (approx. 80% women; mean age 38 years and baseline BMI of 30) consuming one whole Hass avocado/day as part of a hypocaloric diet for 12 weeks supported weight loss, decreased BMI, total body fat (%) and visceral adipose tissue (g) at a similar level as a hypocaloric diet without an avocado/day [51].
- Postmenopausal women—dietary approach #1—unrestricted messaging to increase FV intake vs. messaging to restrict high-fat foods. Sixty-eight overweight and obese women (mean age 57 years) consuming high FV (HIFV) or reducing consumption of high-fat foods (LOFAT) had significantly lower weight and WC after six months compared to baseline, but the reduced intake of high-fat foods lowered body weight considerably more than the high FV diet (Figure 16A,B) [52]. In the HIFV group, ED was shown to be an independent predictor of weight loss.

**Figure 17:**
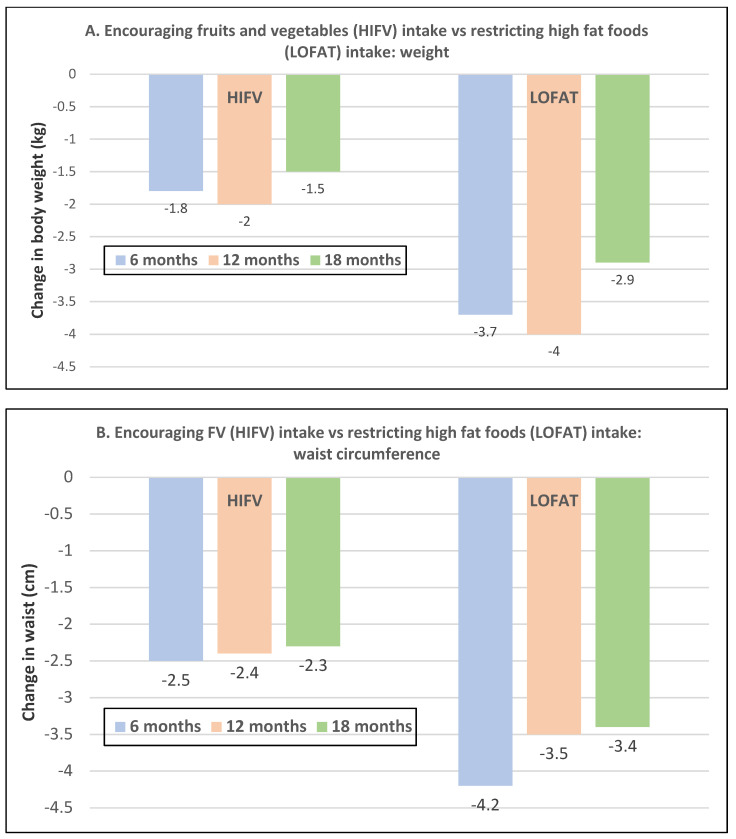
Messaging to encourage FV intake (HIFV) vs. restricting high-fat foods (LOFAT) in overweight or obese postmenopausal women: (**A**) Weight change for HIFV was significantly reduced *p* < 0.05 at 6- and 12-months vs. baseline (*p* > 0.05) but not at 18 months, and the LOFAT group significantly reduced weight at all three times points (*p* < 0.05). (**B**) Waist circumference (WC) change in HIFV group was reduced significantly; WC at 6- and 12-months (*p* < 0.05) vs. baseline (*p* < 0.05) and the LOFAT group had significantly WC decreased at all three time points (*p* < 0.05) [53].

Sixty-eight overweight and obese women (mean age 58 years) who increased their FV intake (HIFV) had a significant or near significant reduction in weight or WC from baseline to over 18 months. In contrast, the low-fat group (LOFAT) had significant weight and WC loss over 18 months vs. baseline that was greater than the HIFV group (Figure 17A,B) [53].

**Figure 18:**
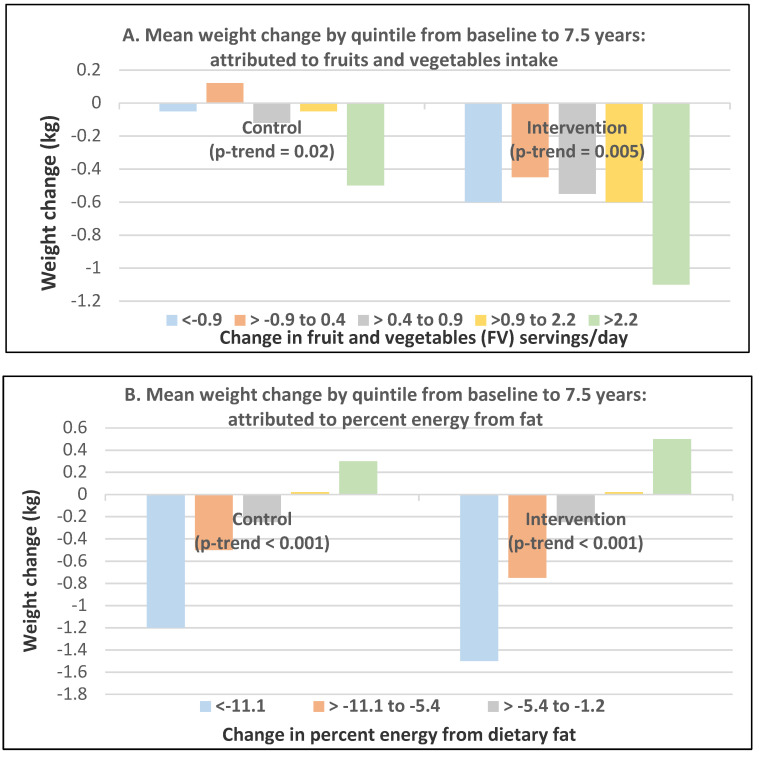
Lower dietary fat and increased fruit and vegetable (FV) (intervention) vs. general dietary guideline information (control) on weight change from baseline to 7.5 years of follow-up that was attributed to (**A**) increasing FV (servings/day) and (**B**) decreasing percent energy from fat in postmenopausal women [56].

- Postmenopausal women—dietary approach #2—active guidance to increase FV intake vs. passive information on general health goals. In US Women’s Healthy Eating and Living (WHEL) Study, 2718 overweight breast cancer women survivors (mean baseline age 53 years) asked in the active guidance group to increase FV intake and lower ED by 25% had weight loss of −0.05 ± 0.12 kg compared to those with passive, general health information guidance who increased weight by 0.71 ± 0.11 kg after one year [54]. However, after four years, the difference in weight between groups was no longer significant. In a Brazilian RCT of 80 adults (76% women; mean age 47 years and BMI of 29), those who increased FV by 109 ± 320 g/day, including 2.9 ± 5.9 g/day FV fiber, decreased weight by −1.4 ± 2.6 kg and BMI by −0.49 ± 1.06 kg/m^2^ (*p* < 0.05) vs. no significant reductions for controls compared to baseline weight after 6 months [55]. An increase of 100 g/day of FV reduced body weight by 500 g and 300 g after six months. The US Women’s Health Initiative Dietary Modification Trial of 48,835 overweight postmenopausal women (mean baseline age 62 years and BMI of 29) intervention included group and individual sessions to encourage reductions in fat intake and increases in FV and grain consumption without adding weight loss or caloric restriction goals [56]. The control group received diet-related education materials. The intervention group women lost weight in the first year (mean of 2.2 kg, *p* < 0.001) and maintained lower weight than control women during 7.5 years of follow-up (difference, 1.9 kg (<0.001 at one year) and 0.4 kg *p* = *0*.01 at 7.5 years). Greater weight loss was associated with an increase in FV servings or a lower intake of % of energy from fat over 7.5 years follow-up. Increasing FV intake showed a significant trend in both groups for more weight loss compared to baseline (Figure 18A). Decreasing percent energy from fat was associated in both groups with more weight loss from baseline (Figure 18B). Also, increasing fiber showed a significant trend toward more weight loss in the intervention women but not in the control women with an increase in fiber by greater than 5.5 g/day, resulting in approximately 1 kg less weight after 7.5 years compared to baseline body weight.

In the USA WHEL Study 52 overweight women breast cancer survivors (mean baseline age 54 years) in the FV intervention group had a marginally significant weight loss of −3.1 kg vs. a gain of 1.6 kg in the passive control group (*p* = 0.07) and similar findings for body fat loss after six months [57]. However, after a follow-up of 12–48 months, the intervention group regained weight to baseline levels while the control subjects showed a relatively stable weight during the follow-up.

**Figure 19:**
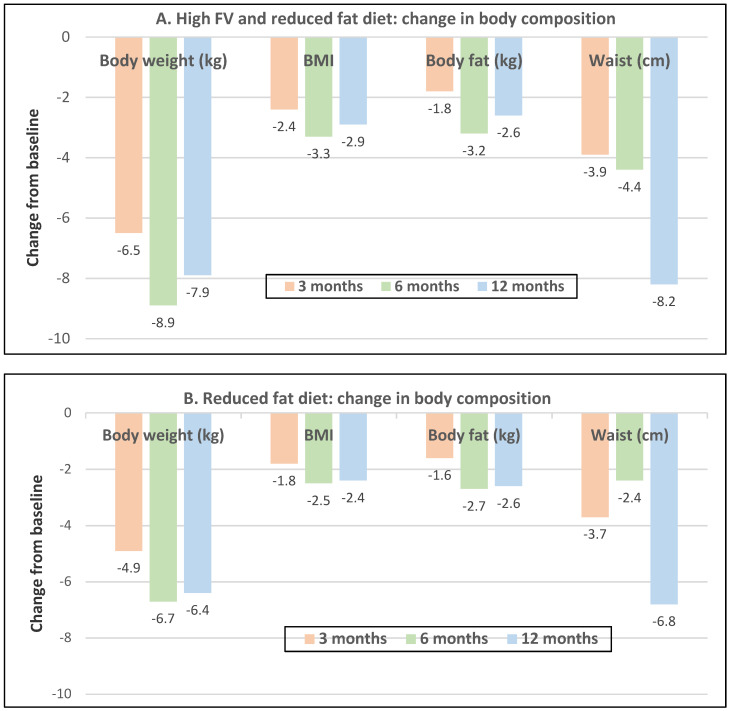
In 71 obese women (mean age 46 years) two ad libitum *diets* were assessed over one year: (**A**) increased fruits and vegetables (FV), and reduced-fat diet reduced weight, BMI and body fat (*p* < 0.0001) and waist circumference (*p* = 0.0002) compared to the (**B**) reduced-fat diet only control diet [58].

**Figure 20:**
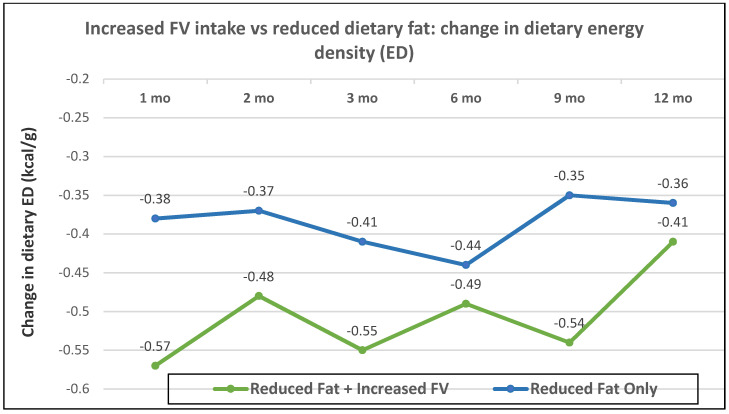
Mean change in dietary ED (kcal/g) from baseline for increased fruit and vegetables (FV) and reduced-fat vs. reduced-fat (control) over 12 months (*p* = 0.019) [58].

- Postmenopausal women—dietary approach #3—reduced dietary fat with and without increased FV with ad libitum amounts of food. Women in the reduced-fat plus increased FV intake group lost 1.5 kg more weight than the reduced fat and no increase in FV intake group (*p* = 0.002) (Figure 19A,B), which was attributed to a significantly lower dietary ED from the combination of both FV and lower fat (*p* = 0.019; Figure 20) [58].

- Postmenopausal women—dietary approach # 4—adding low sodium vegetable juice to Dietary Approaches to Stop Hypertension (DASH) diet. A US RCT in 81 adults (73 women; mean age 50 years) showed that adding low sodium vegetable juice to an energy-restricted DASH diet can aid in weight loss in overweight adults with metabolic syndrome [59]. Overall, in women >45 years, there is probable evidence from RCTs that increased intake of FV can lead to weight loss, especially with concurrent reductions of energy or fat-rich foods. It is also probable that increasing intake of FV alone can contribute to weight stability or modest weight loss that is similar but not as effective as weight-loss diets.

#### 3.3.2. Secondary Study

A two-phase weight loss and weight maintenance RCT showed that adults (63% women, mean baseline age of 56 years) who increased FV intake had an average weight loss of −0.29 kg per 6-months (*p* < 0.0001) in the weight loss phase and −0.04 kg per 6-months in the weight maintenance phase (*p* = 0.0062), per each serving increase [60].

#### 3.3.3. Legume Vegetables

Seven RCTs met the requirements for inclusion as secondary studies assessing the role of FV on body weight and other anthropometric markers in women. Four RCTs in “premenopausal” women, evaluated soy vs. animal or non-soy protein and weight change from 8 to 52 weeks.

##### Premenopausal Women

**Figure 21:**
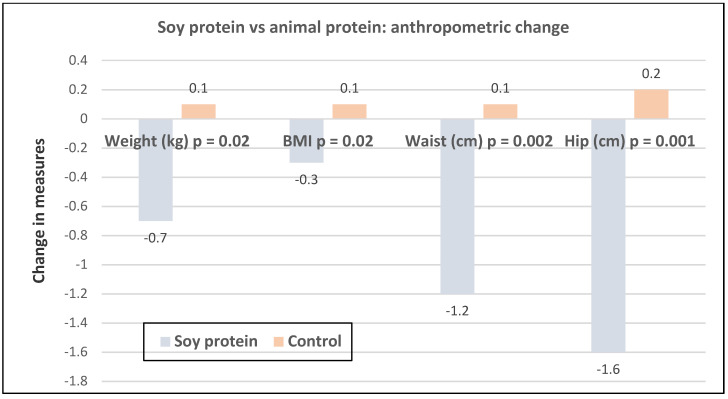
Change in anthropometric measures in 60 women with polycystic ovary syndrome (mean age 25 years and BMI 28) consuming isocaloric diets with 0.8 g protein per kg from soy group (35% soy protein, 35% animal protein and 30% plant protein) vs. non-soy control (70% animal protein and 30% vegetable protein) for eight weeks [61].

A study of premenopausal overweight and obese women (mean age 25 years) consuming non-energy restricted diets with the recommended protein intake of 0.8 g/kg found that premenopausal women replacing 35% of their total protein with soy protein had a significant reduction in body weight, waist, and hip circumferences after eight weeks (Figure 21) [61].

In 58 obese adults (82% women; mean baseline age 42 years and BMI of 33) consuming energy-restricted low fat and high protein diets including three servings/day of soy protein or non-soy protein foods in a group-based weight loss program for four months showed that soy and non-soy protein had similar effects on body weight and fat loss and maintenance (4–12 months) [62].

**Figure 22:**
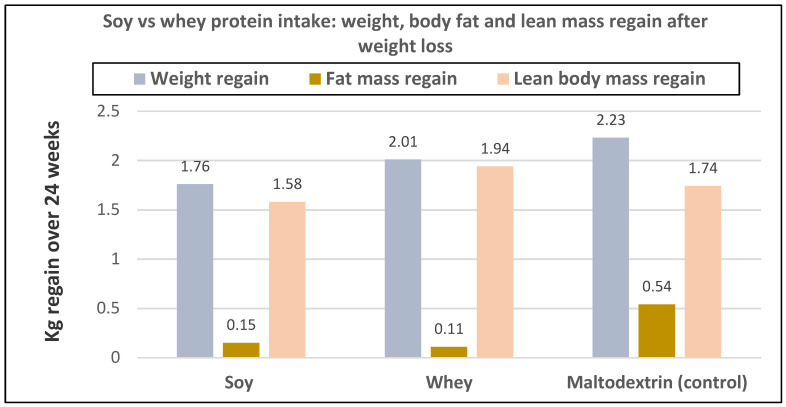
Double-blinded randomized controlled trial on the effects of type of protein (45 g/day) vs. carbohydrate control and weight regain after weight loss; included 171 adults (78% women) over 24 weeks (*p* = 0.50 to 0.96) [63].

A study in overweight and obese adults (78% women; mean age 40 years) showed that in an eight-week weight loss diet followed by a 24-week weight maintenance diet that the isocaloric intake of 45 g/day soy or whey protein resulted in no significant difference between the soy or non-soy proteins or the maltodextrin control in weight regain after weight loss (Figure 22) [63].

In another study, obese women consuming an energy-restricted diet with 15 g/1000 kcal soy protein from soy foods such as veggie burgers and hot dogs, showed no significant differences in % change in weight or body fat compared to women with the same type diet without soy foods after three months [64].

##### Post-Menopausal Women

**Figure 23:**
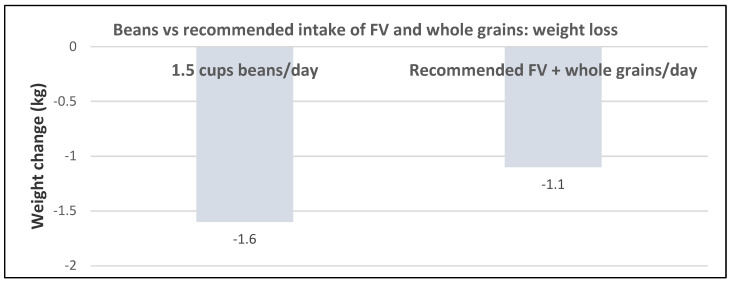
Beans compared to the recommended intake of fruit and vegetable (FV) and whole grains on weight loss in 20 subjects (90% women; mean 47 y and BMI 31) after four weeks (both diets *p* < 0.001; mean fiber 28g/day) [65].

Three RCTs in “postmenopausal” women evaluated the effects of pulses and soy vs. a variety of controls for 4 to 24 weeks. In one study of obese US women, the daily consumption of 1.5 cups/day of beans was as effective as increasing FV and whole grains in promoting weight loss and increasing satiety and reducing hunger (Figure 23) [65].

In another study of 134 overweight and obese postmenopausal Canadian women with metabolic syndrome (mean age 52 years and BMI of 29 ± 5.1), the weekly consumption of 750 mL of pulses did not change body weight or WC compared to an isocaloric diet containing a variety of meats over 16 weeks [66].

**Figure 24:**
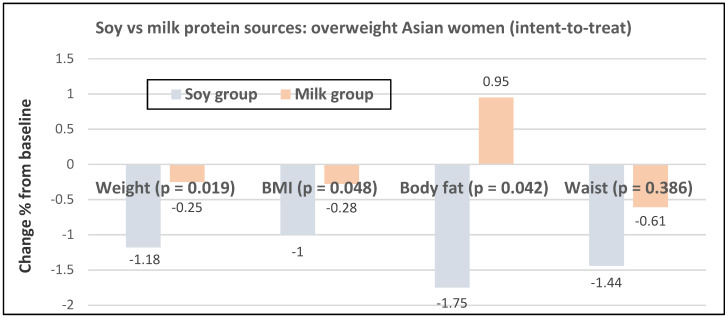
Multivariate % change in anthropometric measures in 180 overweight Chinese women consuming 15-g soy protein vs. milk protein for six months in a double-blind RCT [67].

In a Chinese study of overweight postmenopausal women with pre-diabetes (mean age 56 years), those consuming 15 g of soy protein/day (with 100 mg of soy isoflavones) modestly but significantly reduced % weight and body fat more than those consuming the same amount of milk protein after six months (Figure 24) [67].

### 3.4. Key Mechanisms Influencing Fruit and Vegetable Effects on Weight Loss

In the US, the top 10 consumed FV, their nutrients which may be associated with weight control, and glycemic loads are outlined in Appendix Table A2, Table A3 and Table A4. These values may inform key mechanisms of action.

#### 3.4.1. Dietary Energy Density (ED)

**Figure 25:**
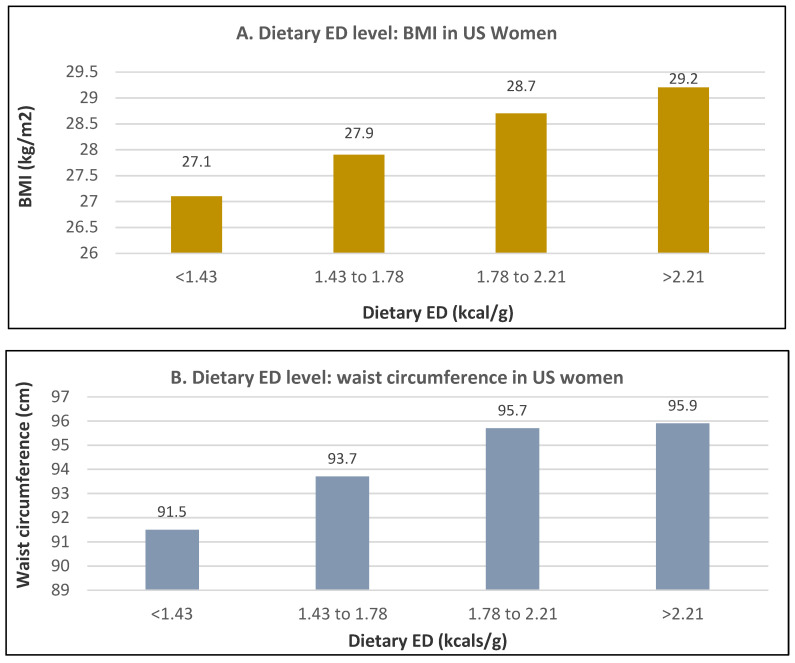
Multivariate adjusted association between dietary energy density (ED) and (**A**) BMI (mean SD ± 0.3 kg/m^2^; *p*-trend < 0.0002) and (**B**) waist circumference (mean SD + mean SD ± 0.9 cm; *p*-trend < 0.001) from an NHANES analysis of 4587 US women (≥18 years) [73].

Dietary ED is one of the most critical factors associated with weight change and the odds of being overweight or obese [68]. High ED diets with large portions of highly processed foods or fast foods are typical of the most common dietary pattern in Western countries, which is higher in percent energy from fat and refined carbohydrates and lower in fiber or bulking volume resulting in rapid energy absorption with nominal impact on satiation and satiety. Low ED diets are rich in whole foods with FV consisting of as much as one-half of each meal as recommended in dietary guidelines throughout the world. Moderate high-fat diets are not necessarily associated with weight gain [2]. For example, the Mediterranean dietary pattern, which is both rich in low ED FV and higher ED olive oil, nuts, and seeds, is associated with reduced weight in women [69,70]. Humans tend to have a relatively low energy regulatory sensitivity to foods or meals with an ED >1.75 kcal/g, which can lead to a positive energy balance and increased risk of weight gain [71]. According to an analysis by the National Health and Nutrition Survey (NHANES), the mean dietary ED of the US diet is 1.9 kcal/g, consistent with the Western diet, which is lower in FV intake, resulting in long-term weight gain [72]. An NHANES analysis of 4587 US women observed a positive linear association between ED and BMI and WC (Figure 25A,B) [73].

A longitudinal study in 186 women (mean age 35.7 years and BMI 26) showed that women consuming higher ED diets (≥1.85 kcal/g), on average, gained 6.4 ± 6.5 kg; women consuming low to moderate ED diets (1.5–1.85 kcal/g) gained 4.8 ± 9.2 kg; women consuming very low to low ED diets (≤1.5 kcal/g) only gained 2.5 ± 6.8 kg after six years [20]. Women in the higher ED group reported consuming significantly more servings from the grain, meats, and fats groups than did the lower ED groups who consumed significantly more servings from FV. The consumption of low ED foods, especially volumetric foods such as most fresh fruits or vegetables, before or as part of a meal, can enhance satiety or reduce hunger and total energy intake during the meal or throughout the day by a mixture of cognitive, sensory, gastrointestinal, hormonal, and neural processes [74,75,76]. Lean women consume more very-low to low ED foods (<1.75 kcals/g) such as salad mix, apples, carrots, green leafy vegetables, citrus fruits, berries, avocados, beans, vegetable soups, low-fat lunch meats, lower fat yogurts and cheeses, eggs, or high protein pasta compared to obese women who consume proportionally more medium to very high-ED foods (>1.75 kcals/g) such as French fries, chicken with skin, natural cheese, full-fat ground beef, potato chips, cookies and candy bars [71,77]. Weight loss maintainers who lost >10% of their body weight and maintained that loss for five years reported that they consumed a mean dietary ED of 1.4 kcal/g compared to those with weight regain who consumed a dietary ED of 1.8 kcal/g [78]. Maintaining energy balance with lower ED foods is especially vital for women who were previously overweight or obese because they had adapted to eating a more substantial amount of food, and consuming low ED foods allows them to continue to consume ad libitum higher volume of food with much lower energy content.

#### 3.4.2. Glycemic Load (GL)

**Figure 26:**
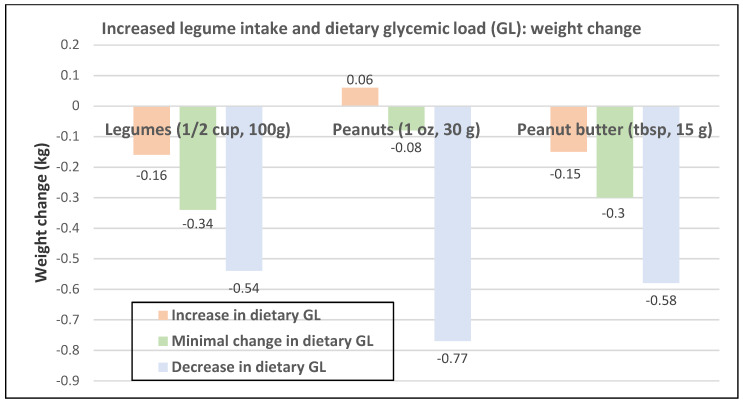
Multivariate adjusted association between increased protein-rich legume vegetable intake per serving/day and dietary glycemic load (GL) status and effect on weight change for all Nurses’ Health Studies (NHS, NHS II), and Health Professionals Follow-up Study (120,784; 80% women) per 4-year period (decreasing GL *p* < 0.01) [19].

Protein foods and carbohydrate-rich foods can interact with each other to affect the trajectory of long-term weight change [19]. Adequate protein intake can support weight control by boosting metabolism and promoting satiation and satiety. In contrast, a high GL causes rises in postprandial blood glucose and insulin, which can reduce the benefits of increased protein foods on physiologic pathways to reduce hunger and weight control. An analysis of three large prospective cohorts of US men and women (80% women) observed that plant protein foods such as legumes (beans, lentils, peas, soy, peanuts, or peanut butter) consumed with higher dietary GL levels were associated with no or a minimal level of weight loss, whereas when they were consumed with lower dietary GL levels were associated with significantly improved weight loss (Figure 26) [19]. Concomitantly, decreasing dietary GL when consuming legume protein foods improves the odds of long-term weight loss compared to increasing dietary GL. A cross-sectional study (809 British women; mean age of 42 years) observed that higher dietary GL increased the odds of general obesity by 91% (*p* = 0.03) and central obesity by 164% (*p* = 0.0008) [79]. Another cross-sectional study in women (*n* = 1113; mean age 55 years) observed that 100 g/day higher intake of FV was negatively associated with visceral fat (−1.6 cm^2^ (95% CI: −2.9 to −0.2 cm^2^; *p* < 0.05) and 100 g/day of sweet snacks was associated with 1.29 fold increase in liver fat content (95% CI: 1.03 to 1.63; *p* < 0.05) [38]. The GL for a range of whole and processed fruits and vegetables are summarized in Appendix Table A1.

#### 3.4.3. Dietary Fiber

**Figure 27:**
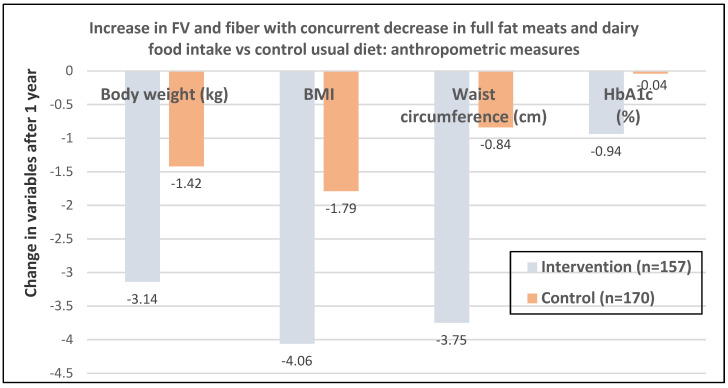
RCT of 327 young overweight and obese women (mean age 34 years). A significant increase in consumption of fruit and vegetable (FV) and fiber along with decreased intake of meat and dairy products, total dietary fat and refined carbohydrates led to reduction of total energy intake by −342 kcals/day in the healthy diet education group resulting in significantly more loss of weight, BMI, and WC than shown in the usual diet control women over 12 months (*p* < 0.05) [81].

**Figure 28:**
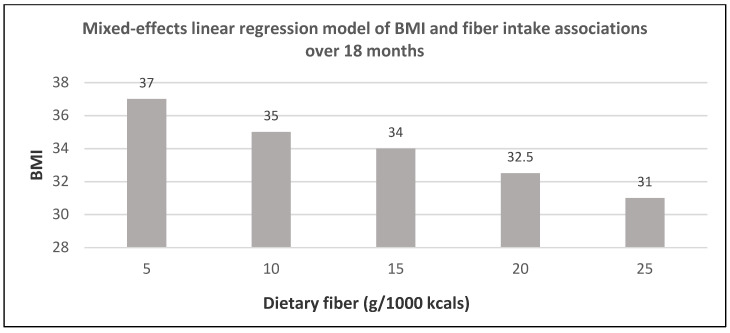
In a randomized weight loss and maintenance trial, increased fiber intake and BMI change estimated over 18 months (6-month intervention and 12 months of follow-up) in middle-aged obese African-American women (*p* = 0.003) [82].

Fiber is associated with several potential weight control and loss mechanisms including (1) lower ED (≤2 kcal/g for fiber vs. 4 kcals/g for refined carbohydrates), (2) increased intestinal bulking volume to promote satiety and satiation, (3) decreased absorption of metabolizable energy from the intestinal tract, and (4) enhanced colonic fermentation and microbiota health to encourage a leaner phenotype profile. FV, including legumes and soy products, provide the majority of fiber in recommended healthy diets followed by whole grains and nuts (Table 1). The POUNDS Lost (Preventing Overweight Using Novel Dietary Strategies) study, a randomized trial in 345 adults (54% women; age 5, BMI of 33) found that of all the dietary predictors, fiber had the strongest association with weight loss with an average increase of 3.7 g/day from baseline, lowering weight by −1.4 kg over six months with no significant interactions between fiber intake and diet type, or sex [80]. In an RCT, 327 young overweight and obese women (mean age 34 years) had a significant increase in daily consumption of fiber by 3.2 g/day, FV by 51.4 g/day, and a decreased intake of meat products, total dietary fat, total energy, and refined carbohydrates [81]. The women in the healthy diet group lost significantly more weight, BMI, and WC than the usual diet control women over 12 months (*p* < 0.05; Figure 27). A US RCT of 101 African-American women (46.4 + 8.4 years and BMI 38.7 + 5.5 kg/m^2^) found that fiber intake was particularly important to help promote weight loss (Figure 28) [82]. Women who had a weight loss of ≥10% of their initial weight had higher fiber intake compared to women who lost <10% or gained weight.

## 4. Discussion

Numerous high quality studies have asked the question of whether consuming FV impacts weight management or weight loss but none of them looked at the totality of evidence for effects by sex. Therefore, this is the first comprehensive critical narrative evaluation of the role of FV in body weight and anthropometric measures focused on women. From the findings, there is probable evidence from prospective and longitudinal studies that increased total FV intake promotes long-term weight stability or loss compared to low total FV intake in women. Total fruit intake tended to have a stronger association with long-term weight stability or loss than total vegetable intake, as starchy vegetables were associated with increased weight over time. There is possible evidence that a serving of starchy vegetables promotes more significantly increased weight gain in older women compared to younger women, which is likely associated with hormonal changes, aging related loss of adipose tissue flexibility, or other metabolic changes (Figure 3). Legumes consumed with low GL diets were more strongly associated with long-term weight loss compared to when they were eaten with high GL diets. The relative weight loss (%) in obese or overweight and normal weight women is generally the same but the net weight loss (kg) tends to be higher in obese of overweight women because of the higher baseline weight.

Data from 12 primary RCTs, six weeks to 24 months in length, also support a role for the increased intake of FV to reduce body weight, BMI, waist, and hip circumference, or body fat in overweight or obese premenopausal women compared to a variety of control diets. Overall, in premenopausal women, there is probable evidence from RCTs that increased intake of FV can lead to weight loss, especially with a concurrent reduction in foods rich in energy or fat. It is also probable that increasing consumption of healthy FV alone can contribute to weight stability or modest weight loss that is similar but not as effective as energy-restricted weight-loss diets. A non-energy restricted diet, including starchy vegetables, fried potatoes, and 100% fruit juice as part of the total FV intake, limits the amount of weight loss. The addition of high amounts of FV and 100% fruit juice to energy-restricted weight loss diets does not tend to change the amount of weight loss compared to weight loss diets low in FV or without 100% juice in women. Eight RCTs using three different dietary approaches consistently support an essential role for the increased intake of FV in preventing long-term weight gains or promoting reductions in weight, BMI, WC and/or body fat in overweight or obese postmenopausal women over six weeks to 7.5 years compared to a variety of control diets. Seven RCTs consistently supported a role for the increased intake of the group of vegetables known as legumes in preventing long-term gains or promoting reductions in weight, BMI, WC and/or body fat in overweight or obese women over eight weeks to one year with non-energy-restricted, weight maintenance, or weight-loss diets.

In obese and overweight women, weight gain occurs if energy intake is higher than energy expenditure [83]. Compared with most other foods, the volume of FV in relation to the energy content is higher. Because of the favorable ED of FV, satiety signals are triggered without consuming a large amount of energy. There are a number of biological mechanism linked to FV regulation of hunger and energy intake for body weight control or loss. Whole FV have a macronutrient nutritional profile that helps to promote neutral or negative energy balance by slowing the rate of eating and increasing satiety. Most FV varieties are considered very-low to low ED, have low GL and little dietary fat content, are good sources of dietary fiber, and an essential source of vitamins, phytochemicals, and minerals [2,83,84,85]. All of these attributes can help promote neutral or negative energy balance to reduce the odds of long-term weight gain and support healthy body compositions and weight loss, especially with concurrent restriction of the intake of high ED or high-fat foods, and increasing fiber intake toward recommended levels [2,68,71,73,83]. Yet, it is crucial to consume the right FVs that can best impact weight control in women (Figure 1, Figure 2 and Figure 5). Fruits or non-starchy vegetables are generally high in bulk volume, less than 100 kcals per serving, and very low or low in ED, mainly because of their physical structure, including high water content and fibrous plant cell walls, and lower fat content and GL [2,84,85]. These FV attributes may assist women in achieving slower eating rates, which were associated with less weight gain or regain after weight loss and lower BMI compared to women with fast eating rates [5]. FV juices (100%) have similar micro-nutritional and phytochemical composition to the whole FV but without the fiber or physical cell wall bulking structure and with a higher GL (e.g., a medium apple has a GL 6 vs. a GL of 10 for unsweetened, reconstituted apple juice; or a medium orange has a GL of 4 vs. a GL of 9 for unsweetened reconstituted orange juice) [85]. Dried FV tend to have similar nutritional composition as whole FV but with higher ED and GL as the water has been removed. An increase of a serving/day of high GL vegetables was not associated with weight change (−0.005 kg) compared to weight loss of −0.15 kg for an increase serving/day of low GL vegetables over 4 years from a study of 86% women [18]. Fried FV are higher in total energy, ED, and fat, which replaces most of the water. Intake of starchy vegetables such as potatoes, especially French fries, or sweet corn, increases the risk of long-term weight gain [17,18]. High fiber vegetables including white potatoes (baked, boiled, or mashed) are associated with no weight change 0.0 kg (−0.09 to +0.09 kg) but excluding white potatoes from higher fiber vegetables is associated with weight loss −0.09 kg (−0.03 to −0.14 kg) per increase serving over 4 years [18]. The top consumed FV do not necessarily contain the more essential nutrients associated with weight control, so there are opportunities to improve the quality of FV consumed.

## 5. Conclusions

This narrative analysis provides a comprehensive and visual assessment of the effects of increasing FV intake and long-term weight change from observational studies and weight loss from RCTs in women. Consistent evidence from prospective studies and RCTs shows that increased intake of FV to recommended levels of intake is a chief contributor to successful weight loss in women. The weight loss effect of consuming FV is enhanced with concurrent dietary restriction of high ED or high-fat foods. Yet, the type of FV differentially impacts weight loss in women. This may be due to the variety of weight loss mechanisms associated with whole FV intake. Contributing factors to weight loss include the ability to eat slow, provide a satisfying very-low to low in ED, GL, and fat content. Also, FV are the primary source of dietary fiber, which provides bulk to the diet and makes people feel full faster thus it helps with weight control when consumed at adequate levels. However, maintaining long-term adequate intake of FV proves challenging, potentially because of a lack of availability of FV, lack of variety in available FV, perceived efforts in preparing FV, perceived cost-benefits, innate evolutionary preferences for salt, fat, and sugar, and confusion around the health benefits of or the recommendations for intake of FV. As men fail to maintain long-term adherence to FV, these challenges are likely not unique to women, but the benefits of consuming FV at recommended levels, may preferentially benefit weight loss in women. As further research is necessary to clarify the role of FV on weight loss, the extensive details provided in this narrative assessment may be used to inform and support the design of future studies.

## Figures and Tables

**Table 1:** Comparison of the dietary fiber content and its food sources for Western vs. healthy diets per 2000 kcals (approx. values) [2,40].

Intake/day	Western Dietary Pattern (US)	USDA Base Pattern	DASH Diet Pattern	Healthy Mediterranean Pattern	Healthy Vegetarian Pattern (Lact-Ovo Based)	New Nordic Diet
Fiber (g)	16	31	29+	31	35+	40+
Fruit (cups)	≤1.0	2.0	2.5	2.5	2.0	2.0 w/berries
Vegetables (cups)	≤1.5	2.5	2.1	2.5	2.5	3–4 w/root vegetables
Legumes (ounces)	--	1.5	0.5	1.5	3.0	1.0+
Soy products (ounces)	0.0	0.5	--	--	1.1	--
Whole-grains (ounces)	0.6	3.0	4.0	3.0	3.0	2.0 +
Nuts/Seeds (ounces)	0.5	0.6	1.0	0.6	1.0	1.0 +

## Appendix A

**Table A1:** Estimated glycemic load (GL) for fruits and vegetables/legumes per serving [18,85].

	Low GL ≤10	Medium GL 11–19	High GL ≥20
Fresh Fruits and 100% Juices			
Avocados	0.1		
Strawberries	1.0		
Oranges, pears, watermelons, tomato juice, grapefruit	3.0–4.0		
Peaches, plums, apricots, blueberries, apples	5.0–6.0		
Grapes, runes	10–11		
Orange juice, apple juice		9.0–10	
Bananas		11–16	
Cranberry juice			16–24
Dried Fruits			
Apricots, mangoes, apples	8–11		
Dates, dried		14–20	
Raisins			28
Vegetables/Legumes			
Peppers, celery, Cauliflower, spinach, kale, iceberg/romaine lettuce, peanuts	0.1–0.2		
String beans, eggplant, zucchini, onions, tomatoes, broccoli, Brussels sprouts	0.4–0.6		
Carrots, cabbage	2.0–3.0		
Winter squash, mix vegetables	3.7–4.4		
Tofu, soybeans soy burger, miso, other soy protein	1.0–5.0		
Beans, lentils, green peas	3.0–9.0		
Sweet corn		11	
Baked/mashed/fried potatoes, sweet potatoes, yams			20–30

**Table A2:** Top ten most consumed whole fruits in the US and their nutrition components per serving, which are generally associated with weight management [84].

Fruits	Serving Size	Total Energy (kcals)	Energy Density kcal/g	Total Fiber (g)	Sugar/Starch (g/g)	Protein (g)	Total Lipid (g)
Edible Portion							
Apples	1 medium (182g)	95	0.5	4.4	19/0.0	0.5	0.3
Avocados	1/3 medium (50g)	83	1.7	3.4	0.15/0.6	1.0	7.4
Bananas	1 medium (118g)	105	0.9	3.1	14 /9.0	1.3	0.4
Blueberries	1 cup (148g)	84	0.6	3.6	15 /0.0	1.1	0.5
Grapes (red or green)	30 grapes (126-g)	87	0.7	1.1	20 /0.0	0.9	0.2
Lemons	1 fruit (58g)	17	0.3	1.6	1.5/0	0.6	0.2
Oranges	1 medium (131 g)	62	0.5	3.1	12 /0.0	1.3	0.2
Peaches	1 medium (147 g)	57	0.2	2.2	12 /0.0	1.3	0.4
Strawberries	1 cup (147 g)	47	0.4	2.9	7.2 /0.1	1.0	0.4
Watermelon	1/16 (280 g)	84	0.3	1.1	17 /0.0	1.7	0.4

**Table A3:** Top ten most consumed vegetables in the US and their nutrition components per serving, which are generally associated with weight management [84].

Vegetables	Serving Size	Total Energy (kcals)	Energy Density (kcal/g)	Total Fiber (g)	Sugar/Starch (g/g)	Protein (g)	Total Lipid (g)
Edible Portion							
Broccoli	1 stalk (148g)	50	0.3	3.9	2.5/2.0	4.2	0.5
Carrots Bag (Baby)	3-oz (85g)	35	0.4	2.0	5.0/0.0	1.0	0.0
Celery	1 medium stalk (40g)	6.4	0.2	0.6	0.5/0.0	0.3	0.1
Lettuce/Mixed Salad Greens	Salad (70g)	10–12	0.14–0.17	0.8–1.4	1.4–0.6/0.0	0.6–1.1	0.1–0.2
Onions	¼ cup (28g)	17	0.6	2.0	4.9/3.0	0.3	0.0
Bell Peppers	1 medium (119g)	29	0.2	2.1	3.6/1.0	1.1	0.3
Potatoes (baked or boiled)	1 medium (167g)	174	1.0	2.8	1.4/28	2.8	4.1
Potatoes (mashed)	1 cup (140g)	190	1.4	2.0	3.0/13	3.0	12
French Fries Fast Foods	1 medium 117g	365	4.5	4.5	0.4/43	4.0	17
Tomatoes Cherry	1 cup (149g)	27	0.2	1.8	4.0/0.0	1.3	0.3
Sweet Corn	1 medium ear (102g)	88	0.9	2.0	6.0/12	3.3	1.4

**Table A4:** Top 10 fruits and vegetables consumed in the US in 2019 [86].

Fruits	Non-Legume Vegetables
Bananas	Potatoes
Apples	Tomatoes
Strawberries	Onions
Grapes	Carrots
Oranges	Broccoli
Watermelon	Bell peppers
Lemons	Lettuce/Salad Mix
Avocados	Cucumbers
Peaches	Celery
Blueberries	Sweet corn

## References

[1] Lee-Kwan S.H., Moore L.V., Blanck H.M., Harris D.M., Galuska D. (2017). Disparities in state -specific adult fruit and vegetable consumption—United States. 2015. MMWR Morb. Mortal. Wkly. Rep..

[2] US Department of Health and Human Services and U.S. Department of Agriculture 2015–2020 Dietary Guidelines for Americans. 8th Edition. December 2015. https://health.gov/dietaryguidelines/2015/guidelines/.

[3] Charlton K., Kowai P., Soriano M.M., Williams S., Banks E., Vo K., Byles J. (2014). Fruit and vegetables intake and body mass index in a large sample of middle-ager Australian men and women. Nutrients.

[4] Williams R.L., Wood L.G., Collins C.E., Callister R. (2016). Comparison of fruit and vegetable intakes during weight loss in males and females. Eur. J. Clin. Nutr..

[5] Karfopoulou E., Brikou D., Mamalaki E., Bersimis F., Anastasiou C.A., Hill J.O., Yannakoulia M. (2017). Dietary patterns in weight loss maintenance: Results from the MedWeight study. Eur. J. Nutr..

[6] Schwingshackl L., Hoffman G., Kalle-Uhlmann T., Arregui M., Buijsse B., Boeing H. (2015). Fruit and vegetable consumption and changes in anthropometric variables in adult populations: A systematic review and meta-analysis of prospective cohort studies. PLoS ONE.

[7] Kaiser K.A., Brown A.W., Bohan Brown M.M., Shikany J.M., Mattes R.D., Allison D.B. (2014). Increased fruit and vegetable intake has no discernible effect on weight loss: A systematic review and meta-analysis. Am. J. Clin. Nutr..

[8] Mytton O.T., Nnoaham K., Eyles H., Scarborough P., Ni Mhurchu C. (2014). Systematic review and meta-analysis of the effect of increased vegetable and fruit consumption on body weight and energy intake. BMC Public Health.

[9] Tapsell L.C., Dunning A., Warensjo E., Lyons-Wall P., Dehlsen K. (2014). Effects of vegetable consumption on weight loss: A review of the evidence with implications for design of randomised controlled trials. Crit. Rev. Food Sci. Nutr..

[10] Arnotti K., Bamber M. (2020). Fruit and vegetables consumption in overweight or obese individuals: A meta-analysis. West. J. Nurs. Res..

[11] Alinia A., Hels O., Tetens I. (2009). The potential association between fruit intake and body weight—A review. Obes. Rev..

[12] Guyenet S.J. (2019). Impact of whole, fresh fruit consumption on energy intake and adiposity: A systemic review. Front. Nutr..

[13] Ledoux T.A., Hingle M.D., Baranowski T. (2011). Relationship of fruit and vegetables intake with adiposity: A systematic review. Obesity. Rev..

[14] Schulze M.B., Fung T.T., Manson J.E., Willett W.C., Hu F.B. (2006). Dietary patterns and changes in body weight in women. Obesity.

[15] Aljadani H.M., Patterson A., Sibbritt D., Hutchesson M.J., Jensen M.E., Collins C.E. (2013). Diet quality, measured by fruit and vegetable intake, predicts weight change in young women. J. Obes..

[16] Aljadani H.M., Patterson A., Sibbritt D., Taylor R.M., Collin C.E. (2019). Frequency and variety of usual intakes of healthy foods, fruit, and vegetables predicts lower 6-year weight gain in young women. Eur. J. Clin. Nutr..

[17] Mozaffarian D., Hao T., Rimm E.B., Willett W.C., Hu F.B. (2011). Changes in diet and lifestyle and long-term weight gain in women and men. N. Engl. J. Med..

[18] Bertoia M.L., Mukamal K.J., Cahill L.E., Hou T., Ludwig D.S., Mozaffarian D., Willett W.C., Hu F.B., Rimm E.B. (2015). Changes in the intake of fruits and vegetables and weight change in the United States men and women followed for 24 years. Analysis from three prospective cohort studies PLoS Med..

[19] Smith J.D., Hou T., Ludwig D.S., Rimm E.B., Willett W., Hu F.B., Mozaffarian D. (2015). Changes in intake of protein foods, carbohydrate amount and quality, and long-term weight change: Results from 3 prospective cohorts. Am. J. Clin. Nutr..

[20] Savage J.S., Marini M., Birch L.L. (2008). Dietary energy density predicts women’s weight change over 6 y. Am. J. Clin. Nutr..

[21] Bes-Rastrollo M., Martínez-González M.A., Sánchez-Villegas A., de la Fuente Arrillaga C., Alfredo Martínez J. (2006). Association of fiber intake and fruit/vegetable consumption with weight gain in a Mediterranean population. Nutrition.

[22] Mirmian P., Bahadoran Z., Moslehi N., Bastan S., Azizi F. (2015). Colors of fruits and vegetables and 3-year changes of cardiometabolic risk factors in adults: Tehran lipid glucose study. Eur. J. Clin. Nutr..

[23] Linde J.A., Utter J., Jeffery R.W., Sherwood N.E., Pronk N.P., Boyle R.G. (2006). Specific food intake, fat, fiber intake, and behavioral correlates of BMI among overweight and obese members of a managed care organization. Int. J. Behav. Nutr. Phys. Act..

[24] He K., Hu F.B., Colditz G.A., Manson J.E., Willett W.C., Liu S. (2004). Changes in intake of fruits and vegetables in relation to risk of obesity and weight gain among middle-aged women. Int. J. Obes..

[25] Wang T., Heianza Y., Sun D., Zheng Y., Ma W., Rimm E.B., Manson J.E., Hu F.B., Willett W.C., Qi L. (2019). Improving fruit and vegetable intake attenuates the genetic association with long-term weight gain. Am. J. Clin. Nutr..

[26] Auerbach B.J., Littman A.J., Krieger J., Young B.A., Larson J., Tinker L., Neuhouser M.I. (2018). Association of 10% juice consumption and 3-year weight change among postmenopausal women in the Women’s Health Initiative. Prev. Med..

[27] Rautiainen S., Wang L., Lee I.-M., Manson J.E., Buring J.E. (2015). Higher intake of fruit, but not vegetables or fiber, at baseline is associated with lower risk of becoming overweight or obese in middle-aged and older women of normal BMI at baseline. J. Nutr..

[28] De Munter J.S., Tynelius P., Magnusson C., Rasmussen F. (2016). Longitudinal analysis of lifestyle habits in relation to body mass index, onset of overweight and obesity: Results from a large population-based cohort in Sweden. Scand. J. Public Health.

[29] Vergnaud A.-C., Norat T., Romaguera D., Mouw T., May A.M., Romieu I., Freisling H., Slimani N., Boutron-Rualt B.-R., Clavel-Chapelon F. (2012). Fruit and vegetable consumption and prospective weight change in participants of the European Prospective into Cancer and Nutrition-Physical Activity, Nutrition, Alcohol, Cessation of Smoking, Eating Out of Home, and Obesity study. Am. J. Clin. Nutr..

[30] Romaguera D., Ängquist L., Du H., Jakobsen M.U., Forouhi N.G., Halkjær J., Feskens E.J., Masala G., Steffen A., Palli D. (2011). Food composition of the diet in relation to changes in waist circumference adjusted for body mass index. PLoS ONE.

[31] Romaguera D., Ängquist L., Du H., Jakobsen M.U., Forouhi N.G., Halkjær J., Feskens E.J., Masala G., Steffen A., Palli D. (2010). Dietary Determinants of Changes in Waist Circumference Adjusted for Body Mass Index—A Proxy Measure of Visceral Adiposity. PLoS ONE.

[32] Buijsse B., Feskens E.J.M., Schulze M.B., Forouhi N.G., Wareham N.J., Sharp S., Palli D., Tognon G., Halkjaer J., Tjønneland A. (2009). Fruit and vegetable intakes and subsequent changes in body weight in European populations: Results from the project on Diet, Obesity, and Genes (DiOGenes). Am. J. Clin. Nutr..

[33] Yuan S., Yu H.J., Liu M.W., Huang Y., Yang Y.H., Tang B.W., Song Y., Cao Z.K., Wu H.J., He Q.Q. (2018). The association of fruit and vegetable consumption with changes in weight and body mass index in Chinese adults: A cohort study. Public Health.

[34] Parisi S.M., Bodnar L.M., Dubowitz T. (2018). Weight resilience and fruit and vegetable intake among African. Public Health Nutr..

[35] Fernstron M., Fernberg U., Hurtig-Wennlof A. (2019). Insulin resistance (HOMA-IR) and body fat (%) are associated to low intake of fruit and vegetables in Swedish, young adults: The cross-sectional lifestyle, biomarkers and atherosclerosis study. BMC Nutr..

[36] Yu Z.M., DeClercq V., Cui Y., Forbes C., Grandy S., Keats M., Parker L., Sweeney E., Dummer T.J.B. (2018). Fruit and vegetable intake and body adiposity among populations in Eastern Canada: The Atlantic Partnership for Tomorrow’s Health. BMJ Open.

[37] Aguiar-Bloemer A.C., Japur C.C., Francisco L.V., Diez-Garcia R.W. (2019). Dietary quality differences between women with and without weight loss in nutritional treatment. Clin. Nutr. ESPEN.

[38] Van Eekelen E., Geelen A., Alssema M., Lamb H.J., de Roos A., Rosendaal F.R., de Mutsert R. (2019). Sweet snacks are positively, and fruits and vegetables are negatively associated with visceral or liver fat content in middle-aged men and women. J. Nutr..

[39] Heskey C., Oda K., Sabate J. (2019). Avocado intake, and longitudinal weight and body mass index changes in an adult cohort. Nutrients.

[40] Poulsen S.K., Due A., Jordy A.B., Kiens B., Stark K.D., Stender S., Holst C., Astrup A., Larsen T.M. (2014). Health effect of the New Nordic Diet in adults with increased waist circumference: A 6-mo randomized controlled trial. Am. J. Clin. Nutr..

[41] De Oliveira M.C., Sichieri R., Mozzer R.V. (2008). A low-energy-dense diet adding fruit reduces weight and energy intake in women. Appetite.

[42] Rodriguez-Rodriguez E., Lopez-Sobaler A.M., Navarro A.R., Bermejo J.M., Ortega R.M., Andres P. (2008). Vitamin B-6 status improves in overweight/obese women following a hypocaloric diet rich in breakfast cereals and may help in maintaining fat-free mass. Int. J. Obes..

[43] Ortega R.M., Lopez-Sobaler A.M., Andres P., Rodriguez-Rodriguez E., Apraricio A., Bermejo L.M., Garcia-Gonzalez L., Basabe B. (2007). Changes in in thiamin intake and blood levels in young, overweight/obese women following hypocaloric diets based on the increased relative consumption of cereals or vegetables. Eur. J. Clin. Nutr..

[44] Ortega R.M., Lopez-Sobaler A.M., Andres P., Rodriguez-Rodriguez E., Apraricio A., Bermejo L.M., Lopez-Plaza B. (2006). Changes in folate status in overweight/obese women following two different weight control programmes based on an increased consumption of vegetables or fortified breakfast cereals. Br. J. Nutr..

[45] Fatahi S., Daneshzad E., Kord-Varkaneh H., Bellissimo N., Brett N.R., Azadbakht L. (2018). Impact of diets rich in whole grains and fruits and vegetables on cardiovascular risk factors in overweight and obese women: A randomized clinical feeding trial. J. Am. Coll. Nutr..

[46] Tanumihardjo S.A., Valentine A.R., Zhang Z., Whigham L.D., Lai H.C., Atkinson R.L. (2009). Strategies to increase vegetable or reduce energy and fat intake induce weight loss in adults. Exp. Biol. Med..

[47] Djuric Z., Poore K.M., Depper J.B., Uhley V.E., Lababidi S., Covington C., Klurfeld D.M., Simon M.S., Kucuk O., Heilbrun L.K. (2002). Methods to increase fruit and vegetable intake with and without a decrease in fat intake: Compliance and effects on body weight in the Nutrition and Breast Health Study. Nutr. Cancer.

[48] Mensinger J.L., Calogero R.M., Stranges S., Tylka T.L. (2016). A weight-neural versus weight-loss approach for health promotion in women with high BMI: A randomized-controlled trial. Appetite.

[49] Crujeiras A.B., Parra M.D., Rodríguez M.C., Martínez de Morentin B.E., Martínez J.A. (2006). A role for fruit content in energy-restricted diets in improving antioxidant status in obese women during weight loss. Nutrition.

[50] Ribeiro C., Dourado G., Cesar T. (2017). Orange juice allied to a reduced-calorie diet resulted in weight loss and ameliorates obesity-related biomarkers: A randomized control trial. Nutrition.

[51] Henning S.M., Yang J., Woo S.L., Lee R.-P., Huang J., Rasmusen A., Carpenter C.L., Thames G., Gilbuena I., Tseng C.-H. (2019). Hass avocado inclusion in a weight-loss diet supported weight loss and altered gut microbiota: A 12-week randomized parallel-controlled trial. Curr. Dev. Nutr..

[52] Lapointe A., Weisnagel S., Provencher V., Begin C., Dufour-Bouchard A., Trudeau C., Lemieux S. (2010). Using restrictive messages to limit high-fat foods or nonrestrictive messages to increase fruit and vegetable intake: What works better for postmenopausal women?. Eur. J. Clin. Nutr..

[53] Lapointe A., Weisnagel S., Provencher V., Begin C., Dufour-Bouchard A., Trudeau C., Lemieux S. (2010). Comparison of a dietary intervention promoting high intake of fruits and vegetables with a low-fat approach: Long-term effects on dietary intakes, eating behaviors and body weight in postmenopausal women. Br. J. Nutr..

[54] Saquib N., Natarajan L., Rock C., Flatt S., Madlensky L., Kealey S., Pierce J. (2008). The impact of a long-term reduction in dietary energy density on body weight within a randomized diet trial. Nutr. Cancer.

[55] Sartorelli D.S., Franco L.J., Cardoso M.A. (2008). High intake of fruits and vegetables predicts weight loss in Brazilian overweight adults. Nutr. Res..

[56] Howard B.V., Manson J.E., Stefanick M.L., Beresford S.A., Frank G., Jones B., Rodabough R.J., Snetselaar L., Thomson C., Tinker L. (2006). Low-fat dietary pattern and weight change over 7 years The Women’s Health Initiative Dietary Modification Trial. JAMA.

[57] Thomson C., Rock C., Giuliano A., Newton T., Cui H., Reid P., Green T., Alberts D. (2005). Longitudinal changes in body weight and body composition among women previously treated for breast cancer consuming a high-vegetable, fruit and fiber, low-fat diet. Eur. J. Nutr..

[58] Ello-Martin J.A., Roe L.S., Ledikwe J.H., Beach A.M., Rolls B.J. (2007). Dietary energy density in the, treatment of obesity: A year-long trial comparing 2 weight-loss diets. Am. J. Clin. Nutr..

[59] Shenoy S.F., Poston W.S.C., Reeves R.S., Kazaks A.G., Holt R.R., Keen C.L., Chen H.J., Haddock C.K., Winters B.L., Khoo C.S.H. (2010). Weight loss in individuals with metabolic syndrome given DASH diet counseling when provided a low sodium vegetable juice: A randomized controlled trial. Nutr. J..

[60] Champagne C.M., Broyles S.T., Moran L.D., Cash K.C., Levy E.J., Lin P.-H., Batch B.C., Lien L.F., Funk K.L., Dalcin A. (2011). Dietary intakes associated with successful weight loss and maintenance during the Weight Loss Maintenance Trial. J. Am. Diet. Assoc..

[61] Karamali M., Kashanian M., Alaeinasab S., Asemi Z. (2018). The effect of dietary soy intake on weight loss, glycaemic control, lipid profiles and biomarkers of inflammation and oxidative stress in women with polycystic ovary syndrome: A randomised clinical trial. J. Hum. Nutr. Diet..

[62] Speaker K.J., Sayer R.D., Peters J.C., Foley H.N., Pan Z., Wyatt H.R., Flock M.R., Mukherjea R., Hill J.O. (2018). Effects of consuming a high-protein diet with or without soy protein during weight loss and maintenance: A non-inferiority, randomized clinical efficacy trial. Obes. Sci. Pract..

[63] Kjølbæk L., Sørensen L.B., Søndertoft N.B., Rasmussen C.K., Lorenzen J.K., Serena A., Astrup A., Larsen L.H. (2017). Protein supplements after weight loss do not improve weight maintenance compared with recommended dietary protein intake despite beneficial effects on appetite sensation and energy expenditure: A randomized, controlled, double-blinded trial. Am. J. Clin. Nutr..

[64] St-Onge M.-P., Claps N., Wolper C., Heymsfield S.B. (2007). Supplementation with soy-protein rich foods does not enhance weight loss. J. Am. Diet. Assoc..

[65] Turner T.F., Nance L.M., Strickland W.D., Malcolm R.J., Pechon S., O’Neil P.M. (2013). Dietary adherence and satisfaction with bean-based high fiber weight loss diet. ISRN Obes..

[66] Gravel K., Lemieux S., Asselin G., Dufresne A., Lemay A., Forest J.-C., Dodin S. (2010). Effects of pulse consumption in women presenting components of the metabolic syndrome: A randomized controlled trial. Mediterr. J. Nutr. Metab..

[67] Liu Z.-M., Ho S.C., Chen Y.-M., Ho Y.P. (2010). A mild favorable effect of soy protein with isoflavones on body composition—A 6-month double-blind randomized placebo-controlled trial among Chinese postmenopausal women. Int. J. Obes..

[68] Rouhani M.H., Haghighatdoost F., Surkan P.J., Azadbakht L. (2016). Associations between dietary energy density and obesity: A systematic review and meta-analysis of observational studies. Nutrition.

[69] Bes-Rastrollo M., van Dam R.M., Martinez-Gonzalez M.A., Li T.Y., Sampson L.L., Frank B., Hu F.B. (2008). A prospective study of dietary energy density and weight gain in women. Am. J. Clin. Nutr..

[70] Goulet J., Lapointe B., Lamarche B., Lemieux S. (2007). Effect of a nutritional intervention promoting the Mediterranean food pattern on anthropometric profile in healthy women from the Quebec City metropolitan area. Eur. J. Clin. Nutr..

[71] Brunstrom J.M., Drake A.C.L., Forde C.G., Rogers P.J. (2018). Undervalued and ignored: Are humans poorly adapted to energy dense foods?. Appetite.

[72] Kant A.K., Graubard B.I. (2005). Energy density of diets reported by American adults: Association with food group intake, nutrient intake and body weight. Int. J. Obes..

[73] Vernarelli J.A., Mitchell D.C., Rolls B.J., Hartman T.J. (2015). Dietary energy density is associated with obesity and other biomarkers of chronic diseases in US adults. Eur. J. Nutr..

[74] National Center for Chronic Disease Prevention and Health Promotion Low-Energy-Dense Foods and Weight Management: Cutting Calories While Controlling Hunger. https://www.cdc.gov/nccdphp/dnpa/nutrition/pdf/r2p_energy_density.pdf.

[75] National Center for Chronic Disease Prevention and Health Promotion Can Eating Fruits and Vegetables Help People to Manage Their Weight? Research to Practice Series; No. 1. https://www.cdc.gov/nccdphp/dnpa/nutrition/pdf/rtp_practitioner_10_07.pdf.

[76] Rolls B.J. (2017). Dietary energy density: Applying behavioural science to weight management. Nutr. Bull..

[77] Vernarelli J.A., Mitchell D.C., Rolls B.J., Hartman T.J. (2018). Dietary energy density and obesity: How consumption patterns differ by body weight status. Eur. J. Nutr..

[78] Raynor H.A., Van Walleghen E.L., Bachman J.L. (2011). Dietary energy density and successful weight loss maintenance. Eat. Behav..

[79] Murakami K., McCaffrey T.A., Livingstone B.E. (2013). Associations of dietary glycaemic index and glycaemic load with foods and nutrient intake and general and central obesity in British adults. Br. J. Nutr..

[80] Miketinas D.C., Bray G.A., Beyl R.A., Ryan D.H., Sacks F.M., Champagne C.M. (2019). Fiber intake predicts weight loss and dietary adherence in adults consuming calorie-restricted diets: The POUNDS Lost (Preventing Overweight Using Novel Dietary Strategies) Study. J. Nutr..

[81] Saffari M., Pakpour A.H., Mohammadi-Zeidi I., Samadi M., Chen H. (2014). Long-term effects of motivational interviewing on dietary intake and weight loss in Iranian obese/overweight women. Health Promot. Perspect..

[82] Buscemi J., Pugach O., Springfield S., Jang J., Tussing-Humphreys L., Schiffer L., Stolley M., Fitzgibbon M.L. (2018). Associations between fiber intake and body mass index (BMI) among African-American women participating in a randomized weight loss and maintenance trial. Eat. Behav..

[83] Boeing H., Bechthold A., Bub A., Ellinger S., Haller D., Kroke A., Leschik E., Muller S., Oberritter H., Schulze M. (2012). Critical review: Vegetables and fruit in the prevention of chronic diseases. Eur. J. Nutr..

[84] U.S. Department of Agriculture, Agricultural Research Service Food Data Central, 2019. www.fdc.nal.usda.gov.

[85] Atkinson F.S., Foster-Powell K., Brand-Miller J.C. (2008). International tables of glycemic index and glycemic load values: 2008. Diabetes Care.

[86] Top 20 Fruits and Vegetables Sold in the U.S. www.PMA.com/content/articles/top-20-fruits-and-vegetables-sold-in-the-US.

